# Neural Organoids and Organoid Extracellular Vesicles for Spinal Cord Injury Repair

**DOI:** 10.34133/research.1463

**Published:** 2026-10-23

**Authors:** Hao Wang, Qirong Zhou, Hao Zhang, Cheng Jiang, Jiacan Su, Han Liu

**Affiliations:** ^1^ Institute of Translational Medicine, Shanghai University, Shanghai, China.; ^2^ MedEng-X Institutes, Shanghai University, Shanghai, China.; ^3^ National Center for Translational Medicine (Shanghai) SHU Branch, Shanghai University, Shanghai, China.; ^4^ Organoid Research Center, Institute of Translational Medicine, Shanghai University, Shanghai, China.; ^5^ Department of Orthopedics, Xinhua Hospital Affiliated to Shanghai Jiao Tong University School of Medicine, Shanghai, China.; ^6^ Department of Biomedical Engineering, School of Medicine, The Chinese University of Hong Kong, Shenzhen, Guangdong, China.; ^7^ Guangdong Basic Research Center of Excellence for Aggregate Science, School of Science and Engineering, The Chinese University of Hong Kong, Shenzhen, Guangdong, China.; ^8^ Suzhou Innovation Center of Shanghai University, Suzhou, Jiangsu, China.; ^9^ Sanming Second Hospital, Sanming, Fujian, China.

## Abstract

Spinal cord injury (SCI) causes long-term disability in part because secondary injury progressively reshapes the lesion microenvironment and restricts axonal regeneration, circuit re-establishment, and white matter repair. Neural organoids are emerging from human pluripotent stem cell-derived disease models into implantable regenerative constructs that may provide structured cellular niches, support host–graft integration, and contribute to remyelination. However, their translation remains constrained by batch variability, limited vascularization, immune-related risk, and long-term safety concerns. In parallel, organoid-derived extracellular vesicles (OEVs) represent a cell-free modality linked to the organoid platform and are more amenable to controlled dosing and repeated administration. However, direct evidence supporting the therapeutic use of neural OEVs in SCI remains limited. OEVs should not be viewed as inherently superior alternatives to conventional extracellular vesicles (EVs); rather, their potential distinctiveness depends on experimentally verifiable links to source-organoid identity, developmental stage, multicellular context, and cargo–function logic. In SCI repair, current evidence should be stratified into direct evidence from neural OEV studies, supportive evidence from SCI-relevant conventional EV systems and related organoid/OEV studies, and testable translational hypotheses. This Review defines neural organoids and neural OEVs as source-linked but mechanistically non-equivalent repair modules, and proposes a mechanism-linked, stage-aware, and quality-oriented framework to guide OEV characterization, benchmarking, and translational evaluation for SCI repair.

## Introduction

Spinal cord injury (SCI) is one of the most disabling types of injury affecting the central nervous system, characterized by high rates of disability, limited regenerative capacity, and a heavy long-term care burden. The World Health Organization notes that there are currently over 15 million people living with SCI worldwide, and a systematic analysis in GBD 2019 also indicates that SCI continues to constitute a substantial global disease burden [1,2]. Although acute-phase decompression, spinal stabilization, and perioperative management are crucial for limiting the spread of primary injury, existing treatments remain unable to substantially improve long-term functional outcomes; the key bottlenecks lie in limited post-injury neural regeneration and the difficulty in effectively reconstructing functional neural circuits [3,4].

In recent years, research into SCI regeneration and repair has gradually shifted from single-pronged protective or replacement therapies toward comprehensive interventions that combine tissue reconstruction with microenvironmental regulation. Neural organoids derived from human pluripotent stem cells can model neural development and are increasingly being explored as implantable regenerative constructs [5]. With the continuous accumulation of evidence regarding in vivo maturation, host integration, and functional integration, neural organoids are gradually evolving from in vitro models into implantable regenerative units, offering new possibilities for tissue bridging and the reconstruction of relay circuits in SCI [6–10].

Meanwhile, extracellular vesicles (EVs) have emerged as a key avenue for cell-free therapy due to their ability to deliver bioactive cargo such as miRNAs, proteins, and lipids, their potential for engineering and modification, and their suitability for repeated administration [11–15]. Building on this, organoid EVs offer a new research perspective for SCI repair. The organoid origin of organoid-derived extracellular vesicles (OEVs) provides a rationale for investigating whether their cargo and biological activity retain information associated with tissue identity, developmental state, and multicellular interactions. However, such source-linked features require direct comparison with relevant conventional EV preparations before OEV distinctiveness can be established [11,16]. To clarify how neural OEVs differ from conventional EVs at the conceptual and translational levels, Table 1 summarizes their distinctions in source definition, developmental-stage linkage, multicellular context, cargo interpretability, quality-control (QC) linkage, manufacturing relationship, and therapeutic positioning. This expansion from “structural transplant units” to “programmable cell-free signaling units” also positions neural organoids and their OEVs as 2 core modules worthy of systematic discussion in SCI regeneration and repair.

**Table 1.** Conceptual comparison between conventional EVs and neural OEVs

Dimension	Conventional EVs	Neural OEVs	Relevance to SCI
Source	Cell-type based	Organoid-derived	Defined source context
Stage	Variably stage-defined	Associated with organoid maturation state	Potential for stage-aware investigation
Cellular context	Relatively simple donor system	Multicellular neural context	Represents multicellular secretory context
Cargo logic	Product-level description	Source state associated interpretation	Supports source–cargo–function investigation
Therapeutic role	General cell-free agent	Candidate niche-conditioning module	Complements organoid graft repair

Accordingly, this Review does not treat neural organoids and OEVs as 2 interchangeable extensions of the same regenerative strategy. Instead, it advances 4 linked propositions for interpreting organoid-based repair in SCI. First, spinal cord organoids used for SCI repair and OEV production require a multilayer identity framework that integrates developmental self-organization, cellular composition, spatial architecture, patterning mechanisms, and physiological function. Second, organoid transplantation and OEV intervention should be interpreted as source-linked but mechanistically non-equivalent repair modules: The former primarily provides structural, cellular, and circuit-reconstructive substrates, whereas the latter is better positioned as a repeatable, exposure-controllable module for microenvironmental conditioning. Third, OEV-related claims should be stratified into direct functional evidence, supportive evidence from related organoid or EV systems, and testable design hypotheses, rather than treated as a uniform evidence base. Fourth, future studies should adopt stage-aware deployment logic and minimum evidence standards that connect source-organoid identity, vesicle cargo, potency-relevant function, and SCI-specific endpoints. Through this framework, we outline how neural organoids and OEVs may be evaluated not only as emerging therapeutic tools but also as coordinated yet non-equivalent modules within a quality-oriented translational strategy for SCI repair (Fig. 1).

**Fig. 1.**
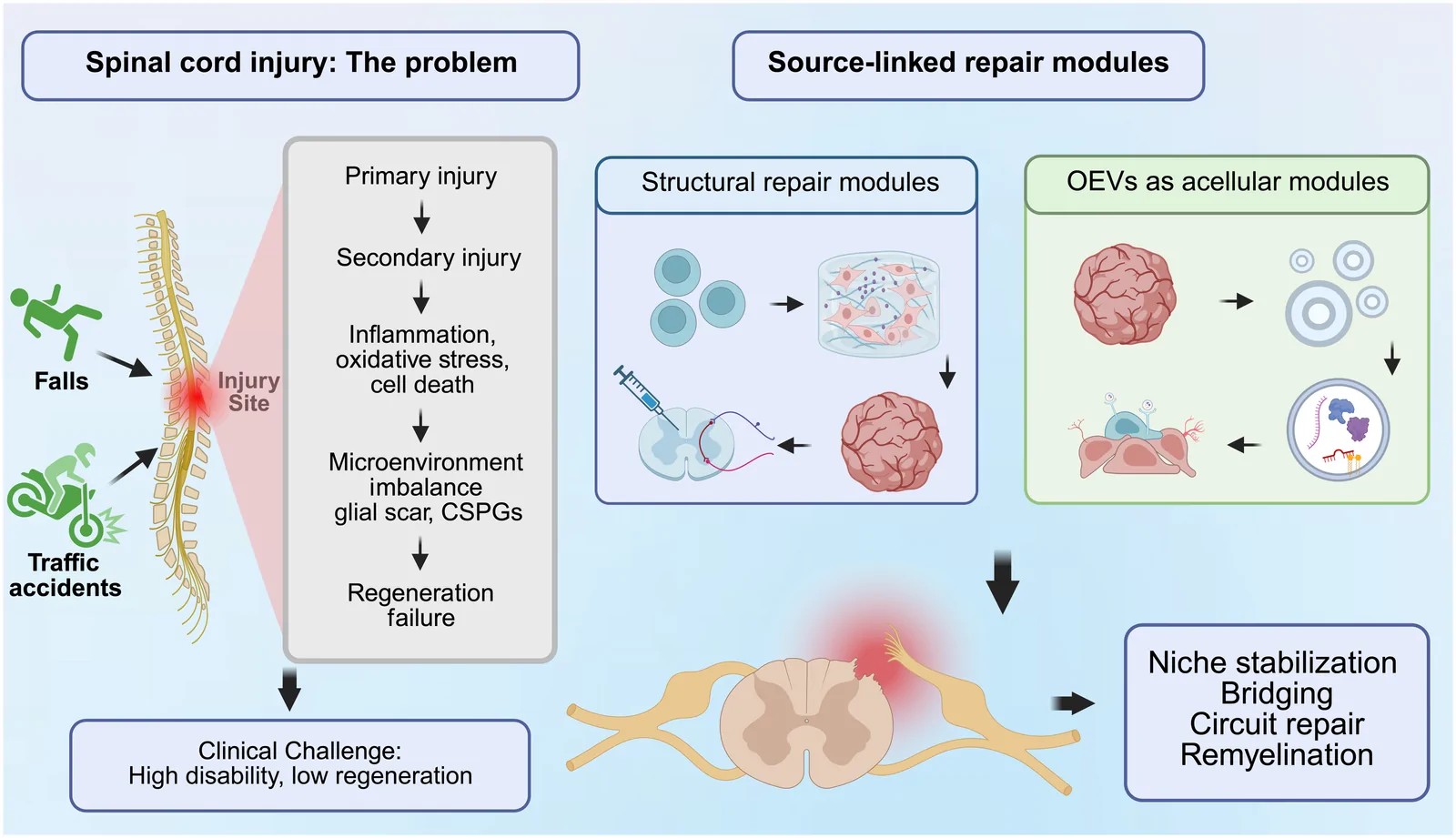
Source-linked but non-equivalent repair modules for SCI. The figure illustrates the pathological progression of SCI and the complementary positioning of neural organoids and OEVs in repair. The left panel shows the injury cascade from primary injury and secondary injury to inflammation, oxidative stress, cell death, microenvironmental imbalance, glial scar formation, and regeneration failure. On the right, neural organoids and OEVs are presented as 2 parallel and complementary source-linked repair modules. Neural organoids serve primarily as structural repair modules for transplantation-based tissue bridging and circuit reconstruction, whereas OEVs act as acellular modules for microenvironmental conditioning. The 2 modules converge on a coordinated repair pathway that collectively supports niche stabilization, tissue bridging, circuit repair, and remyelination.

## Key Pathologies in SCI

The difficulty in repairing SCI does not stem from a single point of failure, but is driven by a combination of multiple pathological obstacles that evolve sequentially. Following primary mechanical injury, local tissues rapidly enter a cascade of secondary damage, manifesting as a series of mutually amplifying pathological processes, including inflammatory activation, oxidative stress, cell apoptosis and necrosis, disruption of ionic homeostasis, and destruction of the blood–spinal cord barrier (BSCB) [17–19]. These changes not only drive the expansion of the lesion area but also cause the local microenvironment to rapidly shift from a relatively plastic state to an imbalanced state characterized by high inflammation, high permeability, and high fragility, thereby substantially narrowing the effective window for endogenous repair and exogenous intervention. Current research generally holds that the limited long-term functional recovery following SCI is not solely attributable to the loss of neurons themselves but is more profoundly constrained by the combined effects of uncontrolled inflammation, barrier disruption, and the continuous deterioration of the tissue microenvironment [3,4].

From the perspective of disease progression, the acute and subacute phases represent critical turning points that determine the outcome of subsequent repair. In the early stages following injury, microglia and peripheral immune cells are rapidly activated and migrate to the lesion site, releasing various inflammatory mediators and reactive oxygen species (ROS), thereby further exacerbating damage to neurons and oligodendrocytes [20–22]; simultaneously, increased BSCB permeability leads to a continuous worsening of peripheral immune infiltration, tissue oedema, and the spread of inflammation. If the inflammatory cascade and barrier instability during this phase are not effectively controlled, this will not only expand the extent of the original damage but also lay the pathological foundation for subsequent scar formation and the deposition of inhibitory matrix [3,4].

As the disease progresses into the subacute to chronic phase, the SCI lesion gradually forms a regenerative-deficient environment characterized by glial/fibrous scar tissue, an inhibitory extracellular matrix (ECM), and residual cavities [23–25]. Reactive astrocytes, fibroblast-like cells, and perivascular cells collectively participate in the remodeling of the injury boundary, accompanied by the continuous accumulation of an inhibitory matrix rich in chondroitin sulfate proteoglycans (CSPGs); simultaneously, myelin-associated inhibitory molecules and a state of chronic inflammation further restrict axonal outgrowth, synaptic integration, and the maturation of the oligodendrocyte lineage. This chronic lesion, characterized by structural disruption, chemical inhibition, and a deteriorating cellular niche, not only hinders damaged nerve fibers from crossing the injury zone but also poses persistent obstacles to remyelination and the reconstruction of functional relay circuits. In other words, the challenge in SCI repair lies not merely in cell replacement or anti-inflammation per se, but in how to simultaneously address the interplay between microenvironmental remodeling, structural bridging, and functional integration [3,4].

For this very reason, effective regenerative strategies for SCI must move beyond interventions targeting a single pathway or a single phase, and instead adopt a multidimensional, comprehensive approach that aligns with the progression of the disease: In the early stages, the aim should be to minimize the inflammatory burden, limit damage to the BSCB, and stabilize the local tissue niche; in the middle and late stages, further support is required for axonal regeneration, synaptic reconnection, remyelination, and the establishment of relay circuits across the lesion [26,27]. These pathological requirements dictate that relying solely on traditional drugs or single-cell therapies often fails to cover the entire SCI repair process, and suggest that future research needs to re-examine regeneration intervention strategies from the dual perspectives of structural reconstruction and microenvironmental regulation.

## Construction and engineering of neural organoids and OEVs

### Spinal cord identity of neural organoids

Although this Review uses “neural organoids” as an umbrella term, SCI-oriented transplantation and OEV production require a more stringent definition of spinal cord-like source identity. Therefore, this section focuses on spinal cord organoids not to narrow the scope of the Review, but to establish the source-identity standard needed to interpret organoid transplantation, OEV cargo, potency, and batch comparability in SCI repair.

Our group’s previous work has supported an integrated view of organoids and their vesicle outputs, underscoring why upstream organoid construction, developmental state, and engineering control should be considered relevant to downstream OEV biology and therapeutic interpretation [28]. In the context of SCI repair, however, the key issue is not only how neural organoids are generated but also how rigorously a neural organoid can be defined as a spinal cord organoid. This distinction is essential because the biological interpretation of organoid transplantation and OEV production depends on whether the source tissue has a reproducible spinal identity rather than a generic neural or neuroepithelial phenotype.

Accordingly, spinal cord organoids should not be defined by isolated neural-marker expression alone. A more stringent identity framework should integrate 5 interconnected levels of evidence: developmental self-organization, cellular composition, spatial architecture, patterning mechanisms, and physiological function. At the level of developmental self-organization, organoids should show neural induction, rosette or neural tube-like morphogenesis, temporal lineage progression, and acquisition of posterior or spinal HOX identity [29,30]. Cellular composition should include spinal-domain progenitors, neurons, interneurons, and glial-lineage cells [31]. At the level of spatial architecture, these cell types should be organized into 3-dimensional (3D) structures with lumen formation, apical–basal polarity, anterior–posterior and dorsal–ventral patterning, segmental identity, and, where relevant, axonal bundles or myelin-associated structures. At the level of patterning mechanisms, these phenotypes should be linked to developmental signaling programs, including RA/WNT/fibroblast growth factor (FGF)-mediated posteriorization, SHH-dependent ventralization, BMP/WNT-related dorsalization, and pathways controlling neurogenesis, gliogenesis, and synaptogenesis. At the level of physiological function, spinal cord organoids should be assessed for neuronal excitability, calcium activity, MEA-detectable network activity, synaptic connectivity, myelination-associated maturation, and, in transplantation settings, host–graft integration and conduction-related recovery [32].

Within this framework, 3D self-organization driven by patterned developmental signaling provides the basis for reproducing the key spatiotemporal coordinate systems of neural tube and spinal cord development in vitro. Axis patterning is typically achieved through the coordinated action of anterior–posterior posteriorization programs, in which RA-, FGF-, WNT-, or GDF-related signals establish spinal and segmental predisposition [33], and dorsal–ventral patterning programs governed by the SHH-BMP/WNT signaling balance. Together, these features provide a practical identity and QC framework comprising HOX segment markers and dorsal/ventral domain markers, such as HOXB4, HOXC6, HOXC9, PAX7, LMX1A, NKX6.1, OLIG2, and MNX1 [5,6,34]. Importantly, such markers should be interpreted as part of a layered identity framework rather than as standalone proof of spinal cord identity [35,36].

Beyond HOX-dependent positional identity, SOX transcription factors provide complementary information on neural progenitor state and regenerative competence rather than spinal segmental identity. SOXB1 factors, particularly SOX1 to SOX3, are involved in maintaining neural progenitor identity and coordinating cell-cycle exit and neurogenesis. For example, SOX1 contributes to preservation of the neural progenitor pool by suppressing Prox1-mediated cell-cycle exit and premature neuronal differentiation [37,38]. In the context of SCI, multiple SOX-family genes have also been implicated in injury-responsive neural stem/progenitor programs and regenerative responses, highlighting their potential relevance to neural repair [39]. Therefore, SOX-associated progenitor-state information may complement, rather than replace, HOX and dorsal–ventral patterning markers when evaluating the developmental identity and maturation state of spinal cord organoids.

Engineering-enabled systems are increasingly important for converting this identity framework into reproducible organoid platforms. Microfluidic gradient neural tube models enable synchronous anterior–posterior and dorsal–ventral patterning within a 3D tubular geometry, providing an engineered approach for controlling developmental baselines and spatial pattern formation [40]. Organoid-on-chip platforms and standardized interfaces with microelectrode arrays further allow time-series electrophysiological readouts [41], including spontaneous activity, burst features, and network-level responses under exogenous stimuli or pharmacological perturbation [42]. Fully defined 3D matrices can also reduce batch-to-batch variation and improve control over organoid size, morphology, and maturation trajectory [43], thereby enhancing reproducibility [44].

For SCI modeling and repair-oriented applications, this layered definition has 2 implications. First, it helps distinguish spinal cord organoids from generic neural aggregates, brain-region organoids, or simplified neural spheroids, thereby reducing ambiguity in transplantation studies. Second, it provides a source-definition standard for OEV studies, because the cargo, potency, and batch comparability of OEVs can only be interpreted meaningfully when the developmental stage, regional identity, cellular composition, and functional maturity of the source organoid are defined. Human spinal cord-derived neural organoids can now accommodate in vitro injury inputs such as laceration and crush trauma, exhibiting immediate neuronal death, glial scar-like tissue formation, and immune-associated responses after immune-cell introduction, thereby establishing a reproducible operational paradigm for closed-loop in vitro SCI modeling [45]. Therefore, upstream organoid construction should be regarded not only as a culture step but also as the source-identity layer that determines how downstream OEV biology, therapeutic interpretation, and translational comparability should be evaluated (Fig. 2). Accordingly, evidence from different neural organoid and neural-lineage systems is not considered interchangeable in this Review. Findings obtained from spinal cord organoids in SCI-relevant settings are treated as the most directly applicable evidence for the spinal organoid module, whereas evidence from brain or other region-specific organoids, neural rosettes, neural stem/progenitor grafts, and isolated OPC transplantation is used as supportive evidence for transferable biological or mechanistic principles. Such extrapolation should be interpreted cautiously because regional identity, developmental state, cellular composition, and maturation may substantially alter graft behavior and EV cargo or function.

**Fig. 2.**
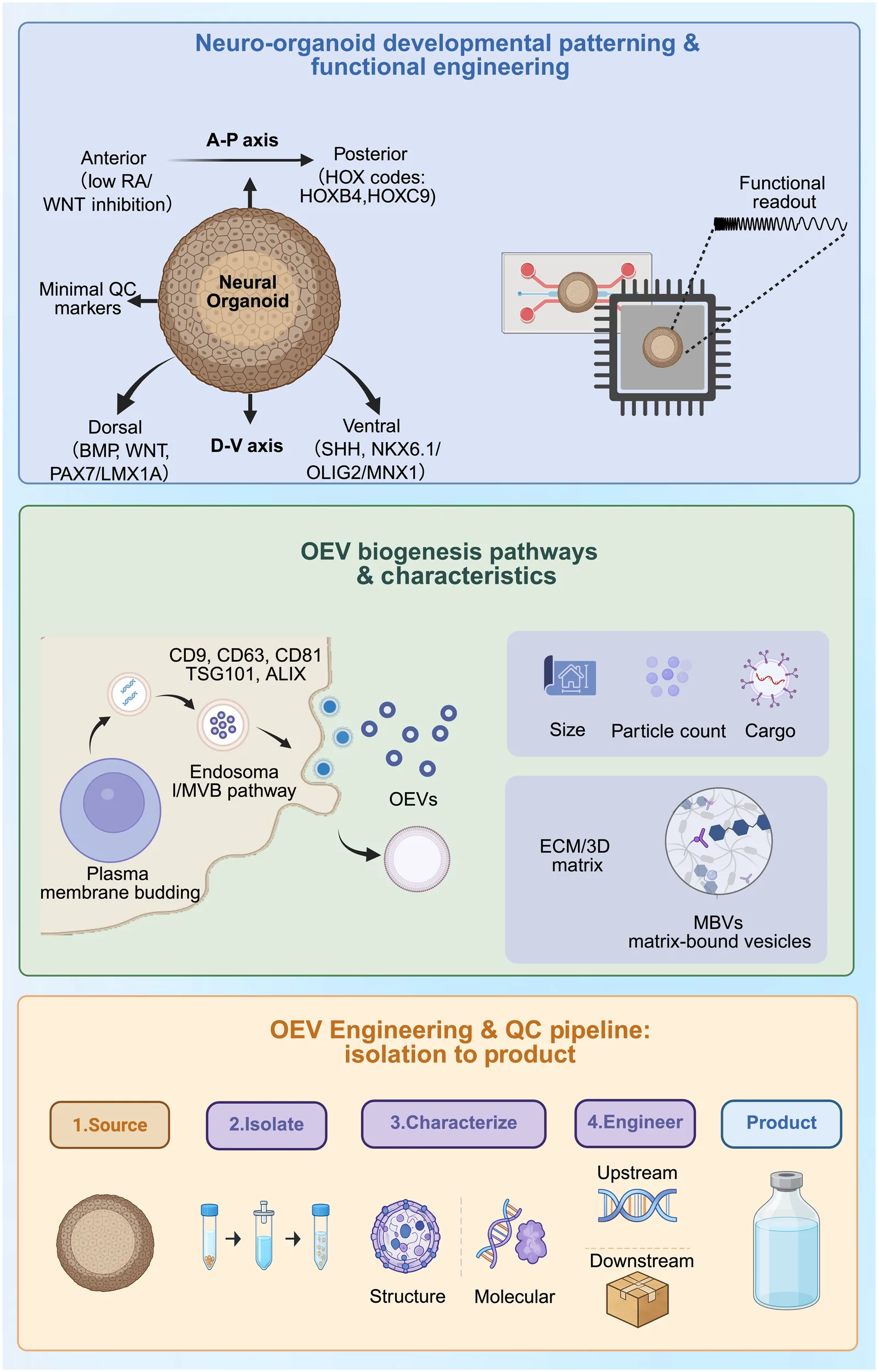
Schematic of neural organoid construction/engineering and the biogenesis and product-oriented workflow for OEVs. The top panel shows developmental signaling-guided 3D patterning that establishes anterior–posterior and dorsal–ventral axes with minimal QC markers, while engineered platforms enable standardized culture and functional readouts. The middle panel shows OEV generation through endosomal multivesicular body-associated release and plasma membrane budding, together with measurable secretory phenotypes and matrix-associated subpopulations. The bottom panel outlines a translational pipeline from pre-clearing, concentration, and purification to orthogonal characterization and controls, integrating source-end or post-isolation engineering and culminating in QC gates and release criteria.

### Biogenesis and product characteristics of neural OEVs

Previous work has discussed the biological principles, therapeutic relevance, and potential distinctiveness of OEVs across organoid systems. This perspective supports the view that neural OEVs should be interpreted not only as generic EVs but also as vesicle products shaped by the developmental and microenvironmental state of the source organoid [46–49].

EVs derived from neural organoids may arise through at least 2 broad formation routes. One involves endosomal multivesicular body-associated release through endosomal sorting and membrane invagination mechanisms. Both ESCRT-dependent and ESCRT-independent mechanisms have been reported, with associated proteins including tetraspanins, TSG101, and ALIX [50]. Another route involves outward budding from the plasma membrane, accompanied by rearrangement of lipid and protein components, dynamic Ca^2+^ changes, and cytoskeletal regulation [50]. Importantly, these formation routes should not be inferred from particle size alone; size-based EV categories are operational descriptors unless biogenesis has been experimentally demonstrated. Therefore, particle size, quantity, surface markers, and cargo spectrum should be interpreted as product characteristics rather than direct indicators of biogenesis [51].

Evidence from organoid and related 3D culture systems suggests that 3D architecture and ECM context may influence EV subpopulation distribution and cargo profiles, potentially through effects on lineage composition, metabolism, and membrane transport. Changes in EV release and miRNA profiles within 3D glioblastoma organoids further support the possibility that the 3D microenvironment influences EV composition and signaling-related outputs [52]. Forebrain organoids derived from Rett syndrome and ASD models have been reported to acquire disease-related cargo during maturation, indicating that EV secretion profiles are influenced by both genetic background and developmental stage [53,54]. In addition, matrix-bound vesicles (MBVs) can also be isolated from the ECM, raising the possibility that organoids contain matrix-associated vesicle subpopulations with distinct delivery properties [55]. Given the potential influence of 3D architecture and ECM context, isolation, purification, and characterization of organoid vesicles should carefully consider both background contamination and bias in subpopulation recovery.

### Isolation, characterization, and engineering of neural OEVs

For engineered applications, EV isolation from neural organoid-explant cultures requires a practical balance among yield, purity, and scalability [56]. In most cases, the workflow includes pre-clearing, concentration, and purification. At the same time, attention should be paid to residual 3D matrix or ECM components, medium-derived background, and contamination caused by organoid fragmentation, which may introduce cellular debris or organelles. Sampling should also be performed within developmentally matched time windows whenever possible. Chromatography-based or flow-through methods are more suitable for scale-up and can improve batch consistency [57], whereas density gradient centrifugation and immunoaffinity approaches generally provide higher specificity but may preferentially enrich certain EV subpopulations. The choice of method should therefore depend on the specific research aim, and key variables—such as organoid culture duration, regional identity, pretreatment conditions, isolation procedure, and storage—should be reported in a standardized manner [58–60].

OEV characterization should distinguish at least 5 complementary dimensions: EV identity, purity, quantity, source-related attributes, and biological potency. EV identity can be supported by morphology and EV-associated marker profiles, whereas quantity should be reported using particle concentration and size-distribution measurements such as NTA or TRPS [61]. Purity assessment should additionally address nonvesicular extracellular material, including residual matrix-derived components, medium-associated particles and proteins, cellular debris or organelles released by organoid fragmentation, and other soluble extracellular contaminants. Blank matrix and medium controls, together with de-vesiculated or equivalent particle/protein controls, are therefore important for distinguishing vesicle-associated signals from nonvesicular background [11,59]. Source-related attributes should be documented separately through the developmental stage, regional identity, cellular composition, and culture state of the source organoid. Importantly, these physicochemical and source-related measurements do not establish biological potency. Potency should be evaluated independently using mechanism-linked functional assays relevant to the intended SCI endpoint, such as inflammatory regulation, BSCB stabilization, axonal growth support, or myelin-supportive activity. Thus, general particle characterization should support EV identity and product comparability, but should not be used as a substitute for functional potency assessment.

On this basis, engineering strategies for OEVs can be broadly divided into 2 categories: source-side engineering and post-isolation modification. Source-side engineering aims to increase vesicle yield or introduce targeting features by modulating the producing cells or organoids [62], for example, through genetic manipulation or defined stimulation conditions [60]. Post-isolation approaches, in contrast, mainly involve surface functionalization with ligands, antibodies, or peptides [63], as well as in vitro cargo loading methods such as electroporation or sonication [64], to improve targeting, stability, and delivery efficiency [60]. Gains must be evaluated concurrently with membrane integrity, immunogenicity, batch-to-batch consistency, and potency readouts, enabling the establishment of a translatable framework for enrichment, potency, and safety assessment [28]. Overall, this section links OEV isolation and characterization with broader EV engineering principles, thereby supporting batch comparability, potency association, and release-oriented QC design.

## Neural Organoid Transplantation for SCI Repair

In the framework proposed here, transplantation literature is used not only to summarize organoid efficacy but also to define what types of evidence are required to support different repair claims in SCI. This is particularly important because organoid transplantation, OEV intervention, and organoid–OEV combination strategies involve different therapeutic substrates, mechanisms, and evidentiary requirements. Therefore, before discussing specific repair mechanisms, we propose a minimum evidence framework for evaluating spinal cord organoid–OEV studies in SCI repair (Table 2). This framework is proposed in this Review as an interpretive and comparative tool and should not be regarded as an established field-wide consensus or formal guideline.

**Table 2.** Proposed minimum evidence framework for spinal cord organoid–OEV studies in SCI repair

Claim being made	Suggested minimum evidence	Overclaim to avoid
Source-organoid identity	HOX identity, dorsal–ventral patterning, spinal-domain cell types, and neural tube-like 3D organization [45]	Calling generic neural aggregates spinal cord organoids
OEV source coupling and distinctiveness	Defined organoid stage, regional identity, EV identity, cargo profile, and comparison with relevant EV controls [46]	Assuming OEV superiority from organoid origin alone
SCI-relevant mechanism	Readouts for inflammation, BSCB integrity, axonal growth, ECM remodeling, or myelin-supportive effects [66]	Inferring mechanism from motor recovery alone
Structural and circuit repair	Graft survival, lineage maturation, axonal tracing, synaptic markers, and conduction-related readouts [79]	Treating axon entry as proof of functional relay reconstruction
Combination and translation	Organoid-only, OEV-only, combination and control groups, plus batch, dose, potency, biodistribution, and safety data [113]	Equating co-delivery or proof-of-concept efficacy with synergy or clinical readiness

### Neural organoid-mediated regulation of the immune microenvironment

The regulation of the immune microenvironment mediated by neural organoids primarily relies on matching the host’s acute-phase immune communication rearrangement window. Single-cell profiling reveals that cellular interactions and receptor–ligand communication between immune cells and endothelial cells, glial cells, and fibroblasts undergo most pronounced reconfiguration at days 1, 3, and 7 post-injury [65]. Vascularization, glial response, and fibrosis signaling exhibit coupled amplification during the acute phase [66]. Consequently, this stage represents both a high-risk period where grafts are susceptible to inflammatory clearance and the most immunologically plastic intervention window [66]. Following implantation of organoids into the lesion site, their 3D architecture and secretory activity may provide local cues that influence the inflammatory microenvironment and contribute to the establishment of a more permissive reparative niche [67]. As supportive evidence from a nonspinal neural organoid system, transplanted brain organoids in a complete transection model yielded stronger axonal regeneration and higher Basso–Beattie–Bresnahan (BBB) motor scores compared with cell-loaded hydrogels. Concurrently, the aggregation of Iba1-positive cells around organoids and their alignment with regenerating axons indicate a localized myeloid response at the graft–lesion interface. However, Iba1 positivity alone cannot distinguish reparative tissue remodeling from persistent inflammatory activation or a graft-associated host response; therefore, additional phenotypic and functional evidence is required before inferring inflammation resolution or a beneficial myeloid state [68]. Furthermore, in an engineered human spinal cord organoid system, organoids loaded onto Matrigel-filled, channelized type II collagen composite scaffolds were associated with reduced inflammatory readouts and improved motor recovery after transplantation into nude mice with complete transection SCI [69]. However, because this study was performed in immunodeficient hosts, the immune-related findings should be interpreted with caution, as the inflammatory context may differ from that of immunocompetent SCI models. It is noteworthy that the SCI immune cascade is not solely determined by myeloid cells; early neutrophil infiltration and subacute lymphoid components also contribute to shaping the inflammatory trajectory [70]. Current organoid studies therefore support myeloid-state changes and inflammatory infiltration as relevant early immune-related endpoints for further mechanistic evaluation (Fig. 3). Taken together, these findings identify immune reprogramming as a plausible early endpoint for OEV-based intervention, particularly because inflammatory amplification, leukocyte recruitment, and myeloid-state transitions are dynamic and diffusible processes. In this setting, OEVs are better interpreted as exposure-controllable immune-conditioning signals that may improve the permissiveness of the lesion niche, rather than as evidence of structural repair.

**Fig. 3.**
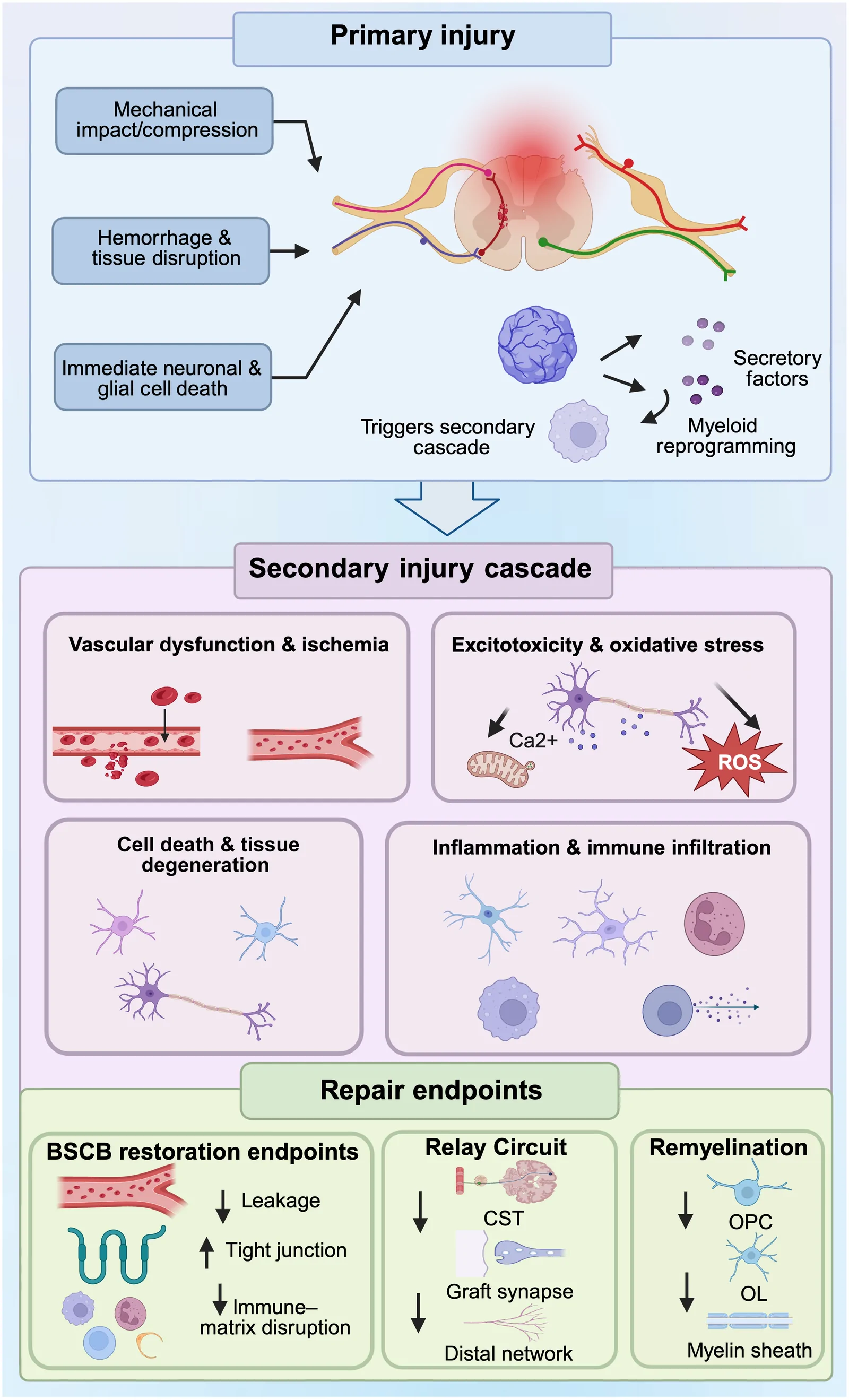
Mechanism-linked endpoint framework for neural organoid-mediated repair after SCI. The upper panel summarizes immediate primary injury events and early neuronal/glial cell loss, together with organoid structural and secretory inputs at the lesion site. The middle panel depicts the evolving secondary injury cascade from minutes to months, including vascular dysfunction and ischemia, edema and cell swelling, excitotoxicity and oxidative stress, inflammation and immune infiltration, and progressive cell death and degeneration. The lower panel summarizes 3 major downstream repair endpoints: (i) BSCB homeostasis, (ii) relay circuit reconstruction, and (iii) remyelination.

### Neural organoid-mediated BSCB recovery

The aforementioned evidence addresses whether the inflammatory response undergoes reprogramming. However, whether inflammation in SCI can genuinely transition from uncontrolled to controlled states is closely linked to the permeability of the BSCB and peripheral infiltration. Suppression of the immune burden and a constructive shift in the myeloid response may influence BSCB-related endothelial damage and peripheral immune infiltration, thereby determining whether the injured region can revert from a highly permeable/highly infiltrated unstable state to relative homeostasis [71]. Therefore, the barrier benefits following neural organoid transplantation should be understood as a chain of evidence that can be directly validated by barrier endpoints [72,73]: firstly, reduced permeability and decreased extravasation [lower leakage of tracers or endogenous immunoglobulin G (IgG), albumin]; secondly, restoration of endothelial junctional integrity (improved expression and membrane localization of tight junction proteins such as Claudin-5, Occludin, ZO-1, and associated adherens junction proteins); thirdly, restricted peripheral immune infiltration (reduced entry of myeloid cells including CD45^+^ leukocytes, Ly6G^+^ neutrophils, and monocytes/macrophages). Regarding the effector pathways, barrier restoration and homeostasis establishment are more likely to sequentially manifest as decreased inflammatory burden and endothelial injury, weakened leukocyte transendothelial migration drive, gradual restoration of the BSCB, and stabilization of the local niche [73]. Suppressing inflammatory and endothelial injury can reduce leakage, edema, and immune infiltration, thereby stabilizing the niche for subsequent reconstruction.

However, the restoration of the BSCB should not be confined to the phenotypic level; it must also provide interpretable structural and molecular foci to elucidate how organoids promote barrier homeostasis at the neurovascular unit level. Structurally, the key lies in the interaction between pericytes and endothelium, alongside the stability of the basement membrane. Given the role of pericytes in maintaining and restoring barrier integrity, this provides a basis for constructing a structural explanatory framework. Organoids indirectly promote pericyte–endothelial coordination and basement membrane stability by reducing local inflammatory burden and enhancing the vascular supportive environment, thereby driving barrier reset and homeostasis maintenance [74]. At the molecular level, barrier-disrupting signaling pathways such as platelet-derived growth factor receptor (PDGFR)-mediated JMJD3 activation can destabilize endothelial junctional architecture and compromise basement membrane integrity, thereby elevating permeability and leakage risk. Inhibiting PDGFR–JMJD3 pathways mitigates BSCB disruption and reduces inflammatory spread, suggesting that suppressing pathways damaging the endothelium and basement membrane constitutes a pivotal mechanism for achieving barrier benefits and maintaining homeostasis [75]. Mechanistic studies further indicate that endothelial junctional instability often involves impaired cytoskeletal anchoring of junctional complexes together with loss or mislocalization of tight junction proteins, leading to diminished barrier function. Consequently, neural organoid transplantation studies should concurrently provide direct barrier readouts such as junctional protein continuity and permeability while distinguishing behavioral improvements from barrier restoration to avoid substituting motor scoring for causal barrier inference [76]. Future research may integrate time-series barrier readouts with mechanistic interventions to further validate causal pathways of barrier benefits. These findings position BSCB stabilization as another early endpoint for OEV-oriented intervention [77]. Because endothelial stress, junctional disassembly, and leukocyte transmigration evolve dynamically after SCI, OEVs may be used to test whether repeated cell-free exposure can preserve barrier-associated permissive conditions. However, barrier readouts should remain distinct from claims of structural reconstruction or circuit repair.

### Reconstruction of relay circuits mediated by neural organoids

Relay circuit reconstruction should be evaluated by whether host axons enter the graft, whether graft-derived neurons form synaptic connections, and whether graft outputs reconnect with host networks distal to the lesion. Previous transplantation studies indicate that post-injury host CST axons can enter the graft even without exogenous guidance, exhibiting a tendency to preferentially connect with motor-related interneuronal domains [78]. This phenomenon suggests that host axons retain the capacity to recognize phenotypically matched targets, thereby providing biological rationale for targeted docking with organoid intrinsic compartments.

Evidence for relay reconstruction can be organized into 3 levels: structural, functional, and causal. Structurally, post-injury host CST axons can enter grafts and show domain-preferential docking, while synaptic marker colocalization and trans-synaptic tracing can further support input–output mapping between host and graft tissues [78–81]. Functionally, in vivo calcium imaging, electrophysiological recording, and sensory-evoked activity responses have shown that grafts can participate in host network activity rather than remaining isolated implants [81–83]. Causally, optogenetic, electrical, or activity-silencing manipulation is required to determine whether graft activity directly contributes to connectivity restoration and behavioral improvement [79,84]. This evidence hierarchy separates anatomical contact from functional coupling and causal relay formation, which is essential for avoiding overinterpretation of axon entry or motor-score improvement alone.

It must be emphasized that even with the aforementioned closed-loop evidence of input, integration, and output, the long-range projection and synaptic integration of organoid axons remain impaired by the inhibitory effects of the injured matrix. Post-injury-enriched CSPGs restrict axonal extension and synaptic integration via the receptor LAR and PTPσ. In human neural progenitor cell transplantation models, inhibiting this signaling axis enhances neuronal replacement and synaptic reconnection while improving functional recovery. This indicates that the CSPG-mediated LAR and PTPσ pathway constitutes a critical external constraint affecting graft axonal topology and integration efficiency [85]. Consequently, enhancing the efficiency and stability of relay circuit formation often requires integrated consideration of organoid identity, compartmentalization, and injury-induced ECM inhibition.

These findings define relay reconstruction as a primarily organoid-dependent function and clarify the nonsubstitutable boundary between organoid transplantation and OEV intervention. OEVs may widen the permissive window for axonal growth and host–graft interaction, but they cannot provide the structured cellular architecture, synapse-forming substrate, or lesion-bridging relay framework required for genuine circuit reconstruction.

### Oligodendroglial and remyelination potential of neural organoids

After neural circuits are re-established, restoration of efficient signal conduction and network synchrony may additionally benefit from white matter repair and remyelination. The remyelinating capacity of oligodendrocyte-lineage cells derived from neural organoids can be evaluated from 2 aspects: whether the organoid can continuously provide OPCs that mature within the SCI microenvironment, and whether these mature cells can then generate myelin sheaths and nodal structures that improve axonal conduction.

Source-side evidence indicates that 3D organoid systems can reconstruct the processes of oligodendrogenesis and myelination [86], forming myelin-like structures and exhibiting node-associated molecular organization. These findings support the intrinsic oligodendroglial and myelinating potential of organoid systems, rather than directly demonstrating functional remyelination after spinal cord organoid transplantation in SCI [87,88]. The critical factor in the injury environment lies in whether maturation and integration occur [89]. As supportive evidence from an isolated oligodendroglial-lineage transplantation system, hiPSC-derived OPC transplantation in SCI models has been associated with enhanced maturation of myelin sheaths and node-associated colocalization, accompanied by electrophysiological and behavioral improvements. Concurrently, endothelial support may amplify remyelination effects in the early stages by enhancing survival and migration [90]. However, findings from isolated OPC transplantation should not be directly extrapolated to spinal cord organoid grafts, because the 2 systems differ in cellular composition, developmental state, and graft architecture.

In the context of chronic scar barriers, pre-OPC transplantation often requires combination with scar management or microenvironment re-engineering to achieve more complete lineage expansion and tissue integration [91]. Consequently, evaluation of organoid oligodendrocyte lineages should prioritize robust evidence combinations, including mature myelin markers, spatial organization of nodal and paranodal proteins, supplemented where necessary by electron microscopy and g ratio, alongside conduction function readouts such as MEP and SEP. This approach avoids relying solely on lineage markers or motor scores to infer remyelination outcomes [90,91]. Taken together, current evidence supports the remyelination potential of the organoid module but does not yet establish functional remyelination after spinal cord organoid transplantation in SCI. The strongest evidence currently derives from the intrinsic oligodendroglial competence of organoid systems and supportive findings from isolated OPC transplantation, whereas direct evidence from spinal cord organoid grafts remains limited. OEVs may plausibly improve the inflammatory, vascular, and metabolic conditions that permit oligodendroglial survival, maturation, and myelin maintenance, and may therefore function as a myelin-supportive microenvironmental module. However, such support remains permissive rather than replacement-level: OEVs cannot by themselves provide a sustained oligodendrocyte-lineage source or directly execute the structural process of sheath formation, paranodal organization, and conduction-restoring remyelination.

Across these 4 mechanistic domains, a consistent pattern of mechanistic boundary emerges between the reparative roles of the organoid and OEV modules in SCI. Neural OEVs may widen permissive windows for repair by modulating inflammatory trajectories, supporting barrier stabilization, reducing matrix-related growth constraints, and conditioning myelin-supportive microenvironments. However, they do not provide a lesion-occupying 3D tissue unit, a sustained lineage source, a synapse-forming substrate for relay reconstruction, or a structurally executing remyelinating compartment. These reparative functions remain more directly attributable to the organoid module. The distinction between organoids and OEVs should therefore be interpreted not as a gradient of the same therapy but as a difference in reparative mechanism, substrate, and deployable function.

### Biomaterial-assisted and donor-cell-engineered neural organoids

The preceding sections define the biological repair functions of neural organoids, whereas the next translational question is how these grafts can be engineered to survive, integrate, and remain controllable in the injured spinal cord. Lesion cavities disrupt tissue continuity and spatial orientation, making it difficult for grafted organoids to integrate in an ordered manner and form relay circuits. They also limit long-term graft survival and recovery of signal conduction by restricting vascular ingrowth and nutrient supply. In parallel, transplant-related inflammation and immune rejection shorten the window for early plasticity and increase the likelihood of graft loss. Two engineering routes are particularly relevant: Biomaterials can organize the lesion–graft interface, whereas donor-cell engineering, including gene-editing approaches, can improve immune compatibility and long-term controllability. Together, these strategies convert organoid transplantation from a cell-placement approach into an interface- and source-controlled regenerative platform (Fig. 4).

**Fig. 4.**
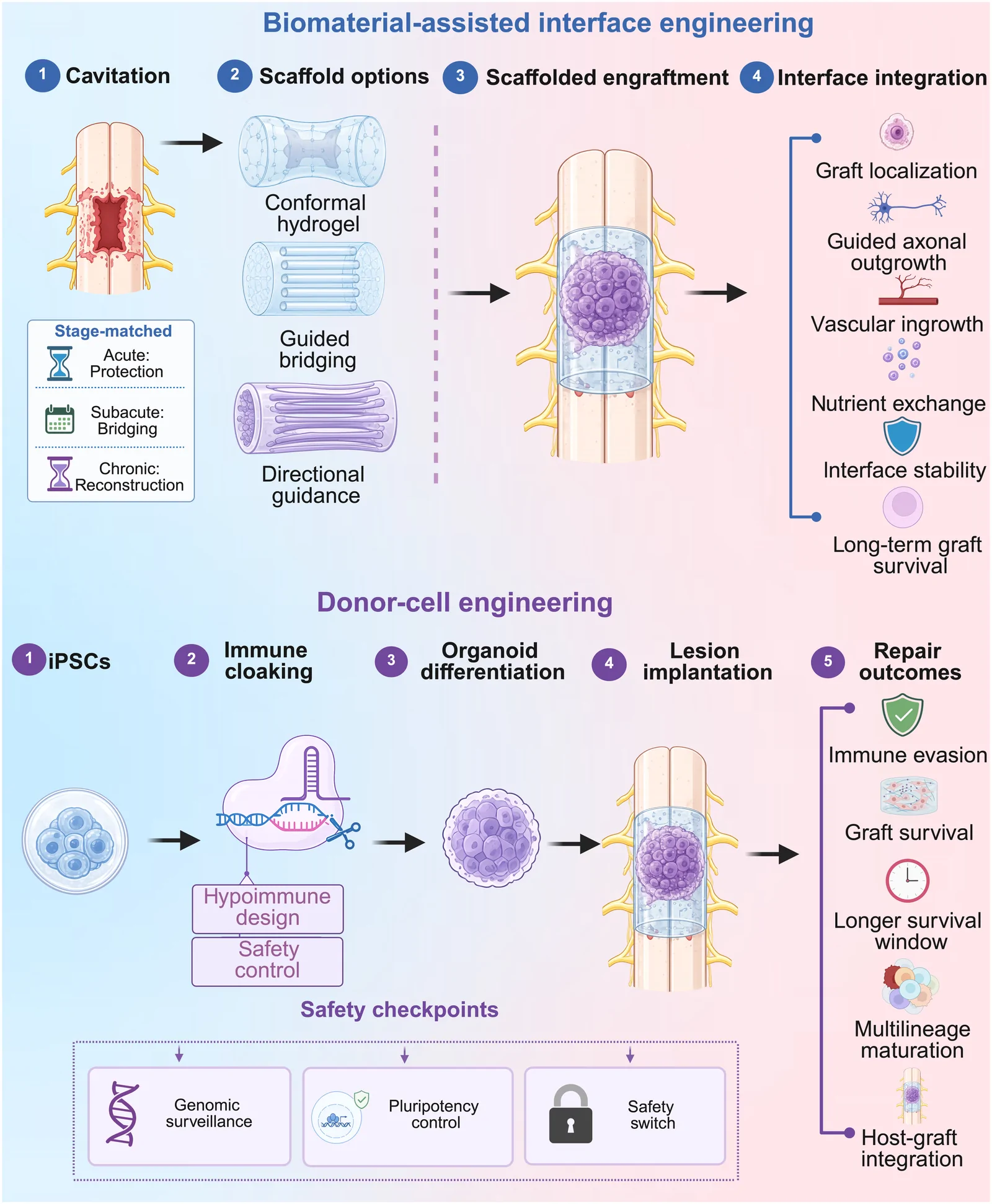
Biomaterial- and donor-cell-engineering strategies to improve neural organoid transplantation. Biomaterial-assisted approaches aim to stabilize graft localization, guide axonal orientation, support vascular ingrowth, and improve the host–graft interface. Donor-cell engineering strategies, including gene editing, may improve immune compatibility and graft persistence, but require genetic stability testing, residual pluripotency control, and safety-switch evaluation.

Engineering strategies should be matched to the dominant barrier at each injury stage. Acute or early subacute lesions require graft protection and immune-stress reduction; subacute cavities require lesion-conformal scaffolds to stabilize graft position and organize the host–graft interface; chronic lesions require anisotropic or channeled templates for spatial alignment, cavity bridging, and interface remodeling.

Biomaterials should be interpreted not as passive carriers but as interface-engineering tools that determine graft localization, axonal orientation, vascular ingrowth, and host–graft boundary stability [92]. Existing scaffold studies collectively support 3 design principles. First, lesion-conformal matrices can improve spatial filling and graft positioning within irregular cavities. Second, aligned or channeled architectures can guide neurite extension and reduce disordered tissue invasion. Third, vascular- or matrix-supportive designs can improve nutrient exchange and increase the likelihood of long-term graft survival. These principles are supported by hydrogel and 3D-printed scaffold studies that organize axonal and vascular structures, guide neurite outgrowth, and improve the controllability of organoid–host reconstruction [93–95]. Accordingly, scaffold selection should be endpoint-driven rather than material-driven. The key readouts should include graft localization and survival, directed axonal and vascular ingrowth, output orientation, perfusion or hypoxia-related exchange, interface stability, and conduction-related recovery, rather than behavioral improvement alone.

Donor-cell engineering provides a second route to improve graft persistence, immune compatibility, and long-term controllability [96]. Evidence from hypoimmunogenic PSC-derived neural-lineage cells and hypoimmune iPSC studies in immunocompetent primates supports the feasibility of reducing alloimmune recognition while preserving long-term survival and multilineage differentiation [97,98]. Specific examples include disruption of B2M and CIITA combined with CD47 overexpression to reduce allogeneic immune recognition, as demonstrated in hypoimmune iPSC models. More recently, hypoimmunogenic iPSC-derived organoids have also been generated, providing organoid-level proof of concept that immune-evasive engineering can be extended to complex 3D tissues [99]. In design terms, hypoimmune engineering is most relevant when long-term graft persistence, repeated transplantation, or allogeneic/iPSC-bank-derived organoids are required. It is particularly important for subacute-to-chronic repair settings, where graft maturation and circuit integration require longer survival windows. However, immune-evasive design should be paired with genetic stability monitoring, residual pluripotency control, and safety-switch considerations. Its benefit should be evaluated through time-series readouts that connect immune infiltration, inflammatory burden, graft maturation, and host–graft integration.

## Repair of SCI by Neural OEVs

### Evidence standards for defining neural OEV distinctiveness

Building upon the source-definition framework outlined above, the central question is not whether neural OEVs appear more complex than conventional EVs, but whether they satisfy the evidence standards required to be interpreted as a distinct and biologically traceable repair module in SCI. In this Review, neural OEV distinctiveness is considered conditional rather than presumed. OEVs should not be separated from conventional EVs merely because they are produced by organoids; instead, their distinction should be supported by an experimentally traceable chain linking source-organoid identity, developmental stage, vesicle cargo, potency-relevant function, and injury-stage-matched deployment. Without this chain, OEVs are better interpreted as complex neural EV preparations rather than as a separate therapeutic class.

#### Evidence stratification for neural OEVs in SCI repair

Because neural OEVs for SCI repair remain an emerging concept, evidence related to this field should be interpreted through a stratified framework. Here, direct evidence refers to findings obtained from neural OEVs themselves, particularly when evaluated in SCI or SCI-relevant models. Because such direct neural OEV–SCI evidence remains limited, findings from SCI studies using conventional cell-derived EVs are considered supportive evidence for transferable mechanistic or delivery principles, including regulation of inflammation, BSCB integrity, lesion enrichment, axonal growth support, and local retention. Supportive evidence also includes findings from neural organoids, organoid EVs, or related neural EV systems that establish biological plausibility, such as source-state-dependent cargo variation, developmental-stage linkage, multicellular secretory outputs, and neurite-promoting activity. Testable hypotheses refer to design concepts derived from these evidence layers but not yet directly validated for neural OEVs in SCI, including stage-matched OEV dosing, cargo-enhanced OEVs, scaffold-associated OEV delivery, and sequential organoid–OEV strategies. This stratification allows neural OEVs to be positioned as a source-linked and engineering-compatible repair concept without overstating them as an already validated SCI therapy.

#### Validation requirements and comparative controls

Building on this stratified framework, 5 validation requirements are particularly important for determining whether neural OEVs represent a source-linked repair module rather than a complex neural EV preparation. First, OEV features must be linked to a definable source-organoid context, including developmental stage, regional identity, cellular composition, culture conditions, and functional maturity. Second, OEV cargo and function should be tested across developmental or regional states of the source organoid, rather than inferred from a single collection time point. Third, OEV-associated cargo should be connected to identifiable SCI-relevant pathological axes, including inflammatory regulation, BSCB stabilization, axonal growth support, remyelination-associated microenvironmental conditioning, and ECM remodeling. Fourth, OEV-associated signals should be evaluated as potential noninvasive readouts of source-organoid state, process drift, and batch comparability. Fifth, any therapeutic claim for OEVs in SCI should be interpreted in relation to injury stage, dosing route, exposure duration, and mechanism-linked endpoints rather than as time-independent vesicle efficacy (Fig. 5).

**Fig. 5.**
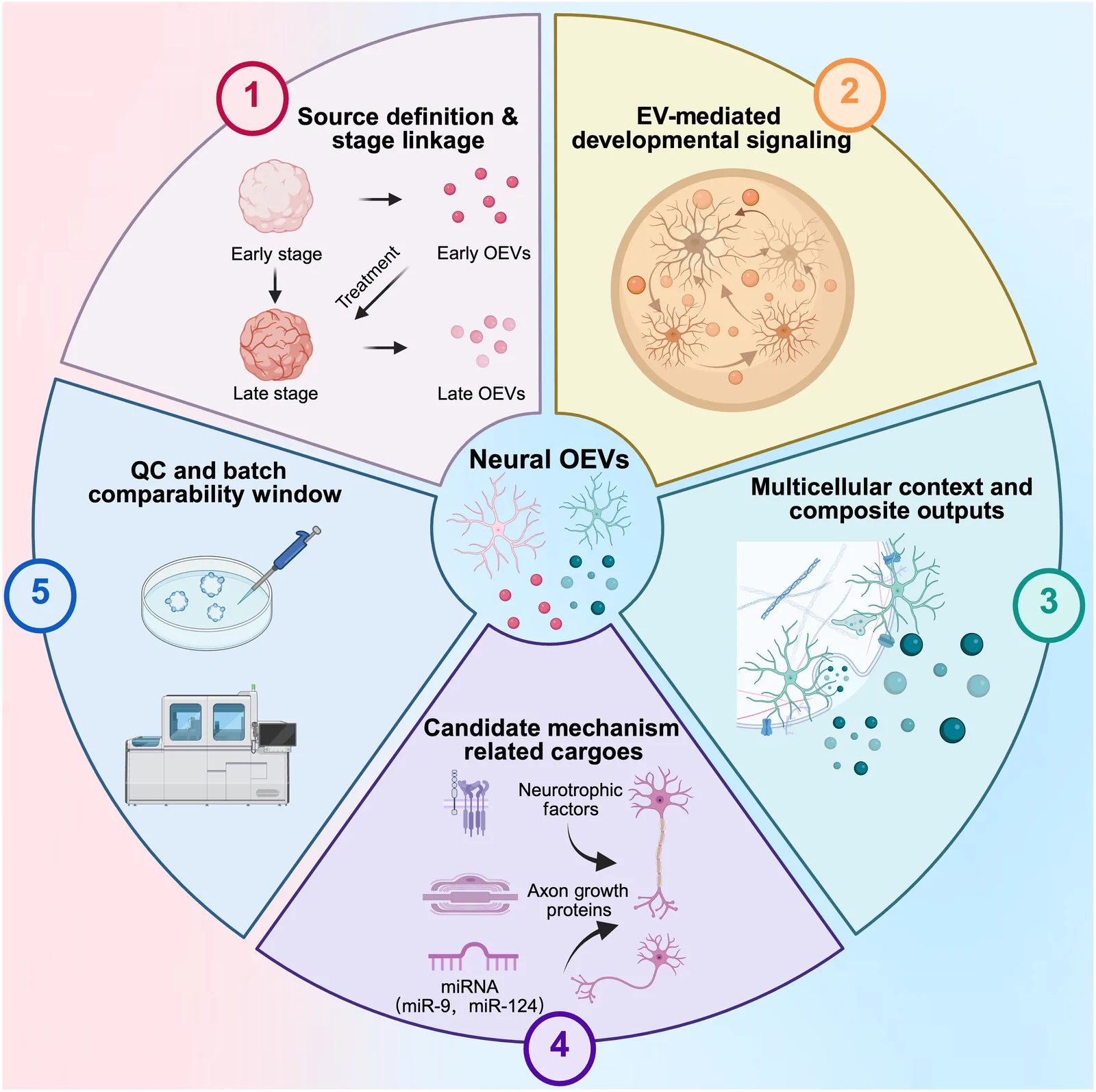
Source-coupled distinctiveness and translational relevance of neural OEVs. The schematic shows how neural OEV properties may be influenced by source-organoid identity, developmental stage, multicellular organization, and culture conditions. OEV profiling may support cargo–function interpretation, potency assignment, and batch comparability, but OEV distinctiveness should be experimentally validated rather than assumed from organoid origin alone.

This evidence standard also requires comparative controls. To establish that neural OEVs provide information beyond generic EV activity, future studies should compare them with relevant EV preparations. These comparisons should be performed under matched experimental conditions, including SCI stage, injury model, administration route, dosing schedule, dose-normalization metric, and endpoint panel. Such comparisons should not be limited to behavioral outcomes, because behavioral improvement alone cannot distinguish microenvironmental protection, axonal growth support, circuit reconstruction, or remyelination. Instead, OEV studies should assign potency to defined endpoint modules, such as immune-cell profiling and cytokine burden for inflammatory regulation, IgG or albumin leakage and tight-junction localization for BSCB stabilization, GAP43/NF200 and tracing-based readouts for axonal growth, and oligodendroglial maturation or conduction-related readouts for myelin-supportive effects. For product development, potency should be defined as a quantitative measure of OEV biological activity linked to the intended mechanism of action rather than inferred from particle number, size distribution, morphology, or marker expression. General characterization assays establish product identity, quantity, and purity, whereas potency assays should demonstrate a reproducible functional effect relevant to the intended SCI endpoint. Mechanistic studies may require broader endpoint panels, but batch release would more realistically rely on a smaller set of qualified, reproducible, and sufficiently rapid functional assays that correlate with the intended mechanism of action. A practical challenge is that many mechanism-linked potency assays depend on cell-based, imaging, barrier-function, or electrophysiological readouts and are therefore more time-consuming and lower-throughput than routine physicochemical characterization. Assay development should therefore balance mechanistic relevance with reproducibility, turnaround time, scalability, and suitability for batch-release testing.

In practical terms, the validation path for neural OEVs can be organized as follows: source-organoid definition → OEV identity and cargo mapping → comparative EV benchmarking → mechanism-oriented potency assignment → injury-stage-matched delivery → endpoint-based therapeutic verification. This sequence is important because it converts OEV research from descriptive vesicle profiling into a testable translational framework in which source, product, timing, function, and disease endpoint are evaluated as a connected system. It also creates a boundary against overinterpretation: Source complexity alone does not prove therapeutic superiority, biodistribution does not prove mechanism-specific targeting, and improved motor scores do not by themselves establish barrier repair, relay reconstruction, or remyelination. In this way, comparative controls convert OEV distinctiveness from a source-based assumption into a testable biological and translational claim.

#### Supportive evidence for source-state-dependent OEV biology

Most current evidence for neural OEV distinctiveness should be interpreted as supportive rather than definitive for SCI, because existing studies mainly support the plausibility of source-state-dependent OEV biology rather than directly demonstrating neural OEV efficacy in SCI. Neural organoid systems can partially reconstruct developmental timing and lineage composition in vitro, making it plausible that their vesicle output varies across developmental stages. For example, EVs released from retinal organoids show stage-specific differences at the small RNA level and can induce transcriptional responses in recipient retinal progenitor cells, supporting the concept that organoid vesicles may encode developmentally dependent information [100]. Within neural organoid systems, EV-mediated transport of developmental cues and inter-domain communication has also been reported, suggesting that EVs may contribute to developmental signal topology rather than functioning merely as passive secretory by-products [101].

Beyond developmental-stage linkage, neural OEV distinctiveness may also arise from the multicellular architecture of organoid and neuroepithelial systems. Because these structures contain interacting neuronal and glial populations, their vesicle output may reflect integrated secretory activity more closely than vesicles derived from simplified donor-cell systems. EVs from neural rosette systems have been reported to contain neuron- and glia-associated components and to display neurite-promoting bioactivity, providing supportive evidence for a secretory phenotype closer to the developing neural microenvironment [102]. These findings support the rationale for investigating neurotrophic, axon-growth-related, and neurogenic miRNA or protein cargoes in SCI repair, but they do not by themselves establish SCI-specific OEV efficacy. Such mechanisms require cargo–function perturbation, such as depletion or inhibition of candidate miRNAs, proteins, or uptake pathways, combined with endpoint-specific rescue or loss-of-function experiments [103,104]

From a translational QC perspective, OEVs may also have value as noninvasive indicators of source-organoid state. Development- or stress-associated nucleic acid signals have been detected in brain organoid EVs, and OEV-associated miRNA profiles may help monitor organoid health and process drift and batch-comparable features relevant to potency hypotheses [105,106]. This possibility is particularly important in the OEV setting because product performance cannot be separated from source-organoid state. If organoid maturation, regional identity, matrix conditions, or stress exposure shift during production, OEV cargo and potency may also drift. Thus, OEV characterization should be linked upstream to source-organoid identity and downstream to potency and release-oriented quality attributes.

Because these studies involve retinal, brain, neuroepithelial, or other nonspinal neural systems, their findings should not be assumed to transfer directly to spinal cord OEVs; regional identity, developmental stage, and cellular composition remain potential determinants of vesicle cargo and function.

Taken together, these studies provide supportive evidence that neural OEV biology may be shaped by source-organoid state, developmental timing, multicellular architecture, and culture conditions [100–106]. However, they should not be treated as direct proof of neural OEV efficacy in SCI. Under the stratified framework proposed above, their main value is to justify testable SCI-oriented designs, including stage-matched dosing, local retention, source-organoid-guided potency assignment, cargo-enhanced OEVs, and sequential organoid–OEV strategies.

### Engineering principles for stage- and microenvironment-matched neural OEV delivery

Based on the evidence stratification described above, neural OEV engineering should be framed as a testable translational strategy rather than assumed to confer therapeutic superiority by source complexity alone. At present, most engineering strategies discussed for neural OEV delivery have been established in conventional cell-derived EV or exosome systems rather than directly validated in neural OEVs. Accordingly, targeting ligands, cargo loading, hydrogel-based retention, and circulation-enhancing modifications are considered here as transferable engineering principles for neural OEVs unless direct OEV-specific evidence is available. Their applicability to neural OEVs therefore requires experimental validation with respect to source-dependent cargo, vesicle properties, potency, and delivery behavior. The key to engineering-driven enhancement of neural OEVs is not only to add targeting ligands or therapeutic cargo but also to match engineering modules with injury stage, microenvironmental barrier, and administration route. In acute SCI, where BSCB disruption, inflammatory amplification, oxidative stress, and rapid clearance dominate, OEV engineering should prioritize lesion enrichment, vascular or barrier targeting, and short-term exposure control. In subacute lesions, where tissue remodeling, glial activation, and early axonal growth windows become more relevant, local retention systems and cargo-enhanced vesicles may be more suitable for sustaining microenvironmental modulation. In chronic or cavity-rich lesions, OEVs are unlikely to replace structural grafts, but may serve as peri-transplant or scaffold-associated modules that improve interface conditioning, matrix remodeling, and graft-supportive exposure. Importantly, the relevance of these engineering strategies in the OEV context lies not only in generic EV enhancement but also in their potential to translate source-coupled biological features into measurable gains in delivery, exposure, and per-dose activity. Surface navigation and entry modules are most useful when the main barrier is insufficient lesion enrichment after systemic or intrathecal administration. For acute and early subacute SCI, peptide- or protein-based targeting strategies may help increase OEV accumulation around injured vasculature, disrupted barrier regions, or activated glial compartments. Taking SCI as an example, Angiopep-2 peptide-modified engineered EVs demonstrate enhanced targeting and enrichment in injury-related regions, delivering reparative benefits and providing a transferable engineering reference for neural OEV targeting rather than direct OEV-specific validation [107]. Such strategies are therefore best viewed as exposure-enhancing modules rather than standalone therapeutic mechanisms. Further multidimensional engineering will couple targeted delivery with immunomodulatory modules such as glial cell reprogramming, presenting a composite effect spectrum encompassing targeting, immune reprogramming, and repair support. This approach may provide a transferable framework for modular OEV design [108].

Cargo engineering is more suitable when delivery alone is insufficient and a defined biological process needs to be amplified. For example, in the subacute repair window, when axonal sprouting, immune resolution, and oligodendroglial support become therapeutic priorities, OEVs may be engineered to carry regenerative peptides, neurotrophic factors, or regulatory RNAs. Exosomes co-loaded with neuron-targeting peptides and regenerative peptides have been shown to enhance axonal regeneration and motor recovery, supporting the feasibility of combining targeting and therapeutic payloads within one vesicle platform [109]. This conventional exosome evidence supports the transferability of combined targeting–cargo design, but its performance in neural OEVs remains to be established. Local retention strategies are particularly relevant when the therapeutic goal is sustained exposure within a lesion cavity or peri-transplant microenvironment. In subacute-to-chronic SCI, where repeated systemic dosing may produce low local exposure and rapid clearance, hydrogel- or scaffold-associated delivery represents a potentially transferable strategy for neural OEVs to convert transient vesicle exposure into a sustained local signal. ROS-responsive hydrogel systems using engineered conventional EVs have demonstrated microenvironment-triggered release, improved lesion retention, and enhanced SCI outcomes [110]. These findings provide supportive engineering evidence for local OEV retention, but direct validation with neural OEVs remains required. These systems are therefore most suitable for cavity-rich lesions, peri-transplant conditioning, or scenarios requiring prolonged barrier, immune, or myelin-supportive modulation. By contrast, systemic exposure-enhancing strategies, such as albumin-binding engineering, may be more appropriate when repeated nonlocal administration is required or when lesions are multifocal, surgically inaccessible, or not suitable for implant-assisted delivery. Albumin-binding engineering has been shown in conventional EV systems to extend circulation time and improve in vivo exposure, providing a transferable pharmacokinetic strategy that may be applicable to neural OEVs [111]. However, its effects on neural OEV biodistribution, cargo integrity, and biological potency remain to be directly validated.

At the conversion stage, engineering choices should be integrated into a product-development loop rather than evaluated only by short-term efficacy. The selected module should match the intended route of administration, expected exposure duration, target lesion stage, and dominant microenvironmental barrier. For instance, hollow-fiber bioreactors support continuous and more homogeneous EV production, providing a process reference for OEV scaling and batch-to-batch consistency [112]. At the same time, engineered heterogeneity, residual materials, altered biodistribution, immunogenicity, and abnormal neuroplasticity should be evaluated alongside potency and exposure readouts [113]. In this way, neural OEV engineering can move from a list of modification strategies toward a stage-matched design logic linking disease phase, delivery route, exposure profile, and mechanism-related potency.

## Combination Strategies of Neural Organoids and OEVs for SCI Repair

Organoid transplantation and OEV intervention should be coordinated according to injury stage rather than treated as interchangeable or automatically synergistic therapies. In this section, we focus on how structurally reconstructive organoid modules and exposure-controllable OEV modules may be deployed sequentially or integratively across acute, subacute, and chronic SCI repair windows. For therapeutic matching purposes, the acute, subacute, subacute-to-early chronic, and chronic phases used here are intended as pragmatic repair windows for aligning dominant pathological barriers with different intervention strategies, rather than as a proposal for a new SCI staging system. Table 3 provides a stage-matched overview of the dominant barriers, proposed modules, key evaluation endpoints, and claim boundaries for organoid–OEV strategies in SCI repair.

**Table 3.** Stage-matched organoid and OEV strategies for SCI repair. The stage-matched modules represent proposed design strategies rather than established stage-specific therapeutic preferences. For neural OEV-containing strategies, current support is derived largely from transferable principles established in conventional EV systems and related organoid/OEV studies; direct validation in neural OEV–SCI models remains limited.

SCI stage	Dominant barrier	Proposed module	Key evaluation endpoints	Claim boundary
Acute	Inflammation, oxidative stress, BSCB disruption	OEVs/engineered EVs	Inflammatory burden; IgG/albumin leakage; tight-junction markers; CD45^+^/Ly6G^+^ infiltration	Motor recovery alone does not prove barrier repair
Subacute	Glial activation, ECM inhibition, axonal growth window	Locally retained or cargo-enhanced OEVs	Lesion retention; exposure duration; GAP43/NF200; CSPG-LAR/PTPσ readouts	Biodistribution does not prove mechanism-specific targeting
Subacute to early chronic	Cavity formation and impaired host–graft interface	Spinal cord organoids ± peri-transplant OEVs	Graft survival; tracing; synaptic colocalization; lineage maturation; electrophysiology	Axon entry does not prove functional relay
Chronic	Dense scar, inhibitory ECM, and poor integration	Scaffold–organoid–OEV platforms	Interface stability; vascular ingrowth; oriented axons; myelin maturation; MEP/SEP	Co-delivery alone does not prove synergy

### Complementary but distinct roles of organoid transplantation and OEV intervention in SCI repair

This stage-matched framework further highlights that organoids and OEVs are complementary but mechanistically distinct repair modules rather than interchangeable therapeutic options.

The relative value of cell-free and graft-based interventions depends on the dominant pathological barrier at each injury stage. Graft-based transplantation can provide a sustained cellular source for tissue reconstruction and circuit rebuilding, but its translation is constrained by surgical invasiveness, early graft loss, limited integration efficiency, immune rejection, and safety risks such as uncontrolled proliferation or ectopic differentiation. These bottlenecks have been highlighted in clinical-grade cell therapy research [114]. Related reviews further emphasize that long-term graft survival, immune compatibility, and safety monitoring remain central constraints for cell- or organoid-based transplantation [115]. By contrast, evidence from conventional EV studies supports the potential utility of repeatable cell-free intervention for tissue protection and microenvironmental conditioning during the acute phase, when secondary injury, vascular instability, and inflammatory amplification dominate. Systematic analyses of stem cell-derived EVs in SCI mainly support tissue-protective, anti-inflammatory, and motor-function-related benefits rather than direct structural reconstruction [116]. Experimental studies using early local or intralesional EV administration further provide a practical reference for acute-window cell-free intervention [117]. These findings provide supportive evidence for acute-window cell-free intervention but do not constitute direct validation of neural OEVs. Neural OEVs may therefore be considered a candidate acute-phase module for microenvironmental conditioning, whose stage-specific utility requires direct validation in neural OEV–SCI models. In the subacute phase, locally retained OEVs may help sustain barrier- and immune-related modulation and prepare the host niche for subsequent integration. When the lesion evolves into tissue discontinuity, cavity formation, and circuit loss, organoid or cell transplantation becomes more relevant because structural bridging, lineage supply, and relay-compatible tissue organization are required. Thus, the key design question is not whether OEVs or organoids are globally superior, but which module matches the stage-specific bottleneck and which endpoints can verify that assignment (Fig. 6). Module selection does not require a single intervention to address every pathological hallmark within a given SCI stage. Instead, the proposed module should be evaluated for sufficient potency against the dominant treatment-limiting barrier for that stage, using mechanism-linked endpoints relevant to that barrier. Additional modules should be introduced only when residual pathological constraints remain likely to limit repair and when the added intervention provides a complementary mechanism rather than redundant activity.

**Fig. 6.**
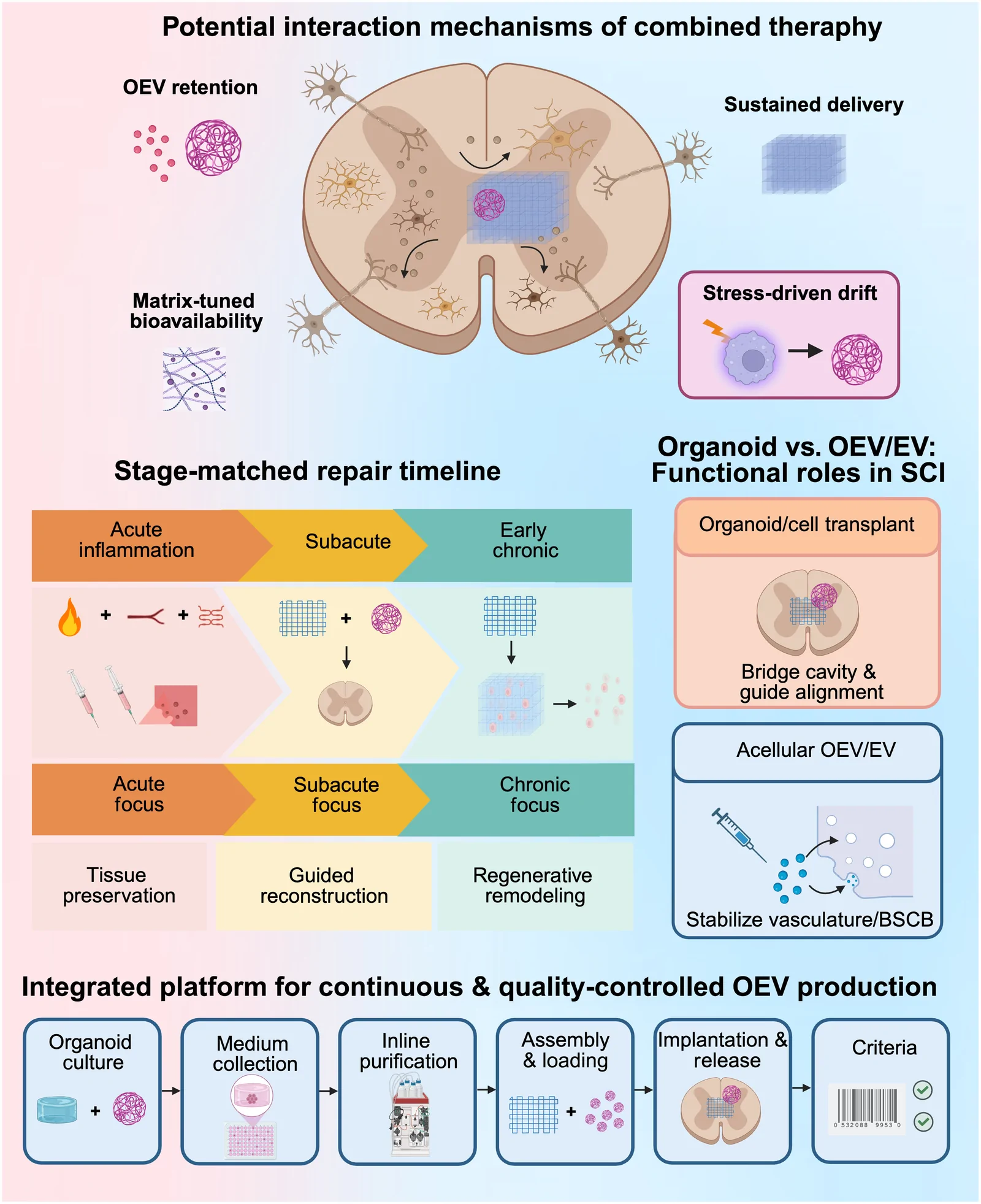
Stage-aware deployment and integrated organoid–OEV platform for SCI repair. The schematic contrasts organoid transplantation as an implantable structural module with OEVs as repeatable, dose-adjustable acellular modules for microenvironmental intervention. A stage-aware strategy is illustrated, prioritizing OEV/EV dosing during the acute inflammatory and barrier-disruption window, introducing organoid transplantation when structural bridging and circuit reconstruction become dominant requirements, and using localized delivery systems to test sustained peri-transplant OEV exposure. The lower panel summarizes an engineered platform for continuous, quality-controlled OEV production and coupled delivery to improve reproducibility and reduce stress-driven drift.

Importantly, such integrated platforms should not be considered intrinsically superior to well-defined target-directed therapies. Their potential advantage lies in the ability to address multiple stage-dependent barriers through complementary structural and microenvironmental modules while still allowing target-specific interventions to be incorporated when appropriate. The anti-Nogo-A experience illustrates the limitation of relying on a single molecular target: In the phase 2b NISCI trial, NG101 did not significantly improve the prespecified upper-extremity motor score in the overall acute cervical SCI population, although post hoc analyses suggested possible benefit in motor-incomplete subgroups. These findings support viewing target-specific therapies as potentially complementary components of a broader stage-matched repair strategy rather than as universally sufficient interventions [118].

For combined organoid–OEV strategies, the critical issue is not to assume synergy from co-administration but to define how an added benefit can be demonstrated, amplified, and translated into reproducible protocols. A plausible interaction mechanism is bidirectional spatial coupling: After organoids establish a local cellular matrix niche, this niche may influence OEV retention, uptake, and diffusion in adjacent tissue, whereas EV–matrix interactions can alter vesicle accessibility and local distribution, thereby affecting whether sufficient signal coverage can be achieved during the peri-transplant period [119–121]. At the delivery level, localized retention and controlled release can convert transient, easily cleared vesicular signals into sustained spatiotemporal gradients. This facilitates the progression of processes such as sustained immune remodeling, axonal growth promotion, and the restoration of vascularization/barrier homeostasis [122–125]. Furthermore, at the biological level, the paracrine output of the transplant itself is expected to extend its effects beyond the graft boundaries to broader tissue areas. This forms a potential bidirectional reinforcement of local structural reconstruction → paracrine diffusion → neighboring cell reprogramming, thereby amplifying local integration advantages into regenerative gains at the neighborhood scale. It must be emphasized that inflammatory and immune stress markedly remodels the biogenesis and cargo profiles of donor-cell EVs, potentially amplifying batch-to-batch variability and reducing predictability [126–128]. Therefore, the integrated system should be designed to regulate processes around the transplantation period with the core constraints of minimizing stress drift and enhancing reproducibility in its temporal organization, rather than merely listing strategies.

### Translational rationale and design considerations for integrated organoid–OEV platforms

Cross-organ studies provide illustrative, but non-equivalent, evidence for the design of integrated organoid–EV platforms. Importantly, evidence from organoid-derived EVs should be distinguished from that obtained using conventional cell-derived EVs. A recent study in the *Journal of Orthopaedic Translation* combined intestinal organoids with intestinal OEVs (IOEVs) for the treatment of IBD complicated by osteoporosis. This approach not only alleviated intestinal inflammation and barrier damage but also reversed the decline in bone mass and bone quality [129]. This approach integrates structural repair units with diffusible signaling units within a single formulation. Its importance lies not in juxtaposing 2 therapies, but in consolidating the entire process—cultivation, production, collection, assembly, and delivery—into a more integrated and potentially controllable workflow. This gives rise to the design concept of an integrated organoid and EV platform. Within this closed-loop system, organoid construction/maturation, continuous in situ production, and online collection of OEVs, coupled with coordinated delivery with implant materials, integrate the structural advantages of cell therapies with the programmable microenvironment benefits of acellular therapies into a more quality-aware and translationally tractable engineered platform. Upstream processes typically use porous scaffolds, hydrogels, or microfluidic perfusion chambers to support organoid culture. These systems help maintain nutrient and oxygen exchange, reduce the risk of necrosis, and allow continuous collection of conditioned medium for EV enrichment. Microfluidic-ECM culture environments have also been reported to improve the structural and functional maturation of human brain organoids, offering useful guidance for integrated strategies that combine organoid cultivation with EV production [130]. At the downstream stage, an important goal is to integrate EV recovery directly into the culture process. By incorporating closed enrichment and purification modules into the perfusion circuit, EV collection can shift from offline batch handling to a more continuous workflow, which may reduce processing loss and improve batch-to-batch consistency. Anion exchange chromatography has been explored as a scalable downstream strategy using HEK293 cell-derived EVs [131]. This example provides a transferable processing reference, but does not directly validate its applicability to organoid-derived EVs. At the “coupled delivery” level, the platform emphasizes co-carriers and co-timing rather than simple mixing: For instance, organoids are stably positioned within scaffold pores/hydrogel networks, while a perfusion system continuously introduces EV-enriched components, establishing a locally sustained concentration background or gradient for EVs. Alternatively, EVs can be pre-loaded onto gel/scaffolds during assembly, creating a coordinated peri-transplant microenvironment of “organoid-stable sites + continuous EVs supply”. For scaling, the platform must integrate scalable culture apparatus to enhance tissue-derived EV yields and reduce process variability. By contrast, a vertical-wheel bioreactor workflow has been described for the production of EVs directly from human blood vessel organoids, providing an organoid-derived EV manufacturing example [132]. More crucially, this co-formulation of structural repair units and diffusible signaling units fundamentally addresses a shared pathological constraint. It necessitates both an implantable 3D structure to occupy space, guide cell migration, and support cellular niches, alongside repeatable vesicular signaling to continuously program the local microenvironment. SCI exemplifies this constraint: Damaged cavities and disoriented axons necessitate structural bridging/ordered alignment, while acute/subacute inflammation and glial reactions demand sustainable, modulatable microenvironmental remodeling. Thus, neural organoids and their OEVs may be regarded as source-linked and potentially integrable outputs of the same organoid platform. The former provides implantable structures and cellular niches, while the latter delivers diffusible, repeat-dosing programming signals. For SCI-targeted integrated organoid–OEV platforms, a co-vector + co-timing approach should lock structural bridging and microenvironmental programming within a single closed-loop process, supporting a stage-matched and testable combination strategy.

To translate this stage-matched platform concept into a testable preclinical strategy, evaluation should follow an iterative rather than strictly sequential workflow. Initial in vitro studies should establish source identity, product quality, mechanism-linked potency, and basic safety criteria sufficient to define a testable candidate, rather than requiring exhaustive in vitro characterization before animal testing. In vivo models should then evaluate lesion-site exposure, biodistribution, host responses, efficacy, and safety, with these findings feeding back to refine in vitro potency assays and candidate selection. Model choice should be question-dependent: Immunocompetent models are important for assessing host inflammatory and immune responses, whereas immunodeficient models can support initial evaluation of human graft survival and integration but are limited for immunocompatibility studies. Humanized models may provide additional value when human-specific immune recognition or hypoimmune engineering is central to the question, while larger-animal models may subsequently be required to address anatomical scale, delivery feasibility, dose translation, and long-term safety.

Beyond biological and delivery integration, future combination strategies may also need to consider how preserved or reconstructed neural circuits are functionally recruited during rehabilitation. In this context, brain–computer interfaces (BCIs) and brain–spine interfaces may provide an activity-dependent complement to organoid–OEV-based repair [133,134]. Organoid transplantation may provide a structural substrate for circuit reconstruction, whereas BCI-mediated rehabilitation may facilitate activity-dependent recruitment and training of residual or reconstructed circuits [133,134]. OEVs could potentially complement this process by conditioning the lesion microenvironment and supporting inflammatory regulation, barrier stabilization, axonal growth, and myelin-associated responses, thereby creating a more permissive environment for activity-dependent plasticity. However, direct evidence that neural OEVs facilitate BCI-mediated rehabilitation is currently lacking. Therefore, the integration of OEVs with BCI should be regarded as a testable multimodal strategy rather than an established therapeutic synergy.

Although developed for SCI, this framework may be transferable to other neuroinflammatory diseases at the level of decision logic rather than through direct reuse of the same therapeutic modules. The principles of identifying the dominant pathological barrier, selecting a mechanism-matched intervention, and validating it with disease-relevant potency endpoints could be retained, whereas the relative roles of organoid and OEV modules would need to be redefined for each disease. For example, in multiple sclerosis, where immune dysregulation and demyelination are major pathological features, EV-based cell-free approaches may be more relevant for immunomodulatory or myelin-supportive intervention [135]. In parallel, organoids may initially have greater value as human disease-modeling and source platforms than as structural grafts in this setting [136]. Accordingly, application of this framework beyond SCI will require disease-specific adaptation and experimental validation rather than direct extrapolation.

## Key Challenges and Countermeasures in Clinical Translation

### Neural organoids: Translational challenges and countermeasures

Neural organoids, as 3D developmental systems derived from induced pluripotent stem cells (iPSCs), first encounter challenges to reproducibility arising from the combined effects of source heterogeneity and process variability. Minor differences in iPSC genotype, reprogramming and expansion history, and critical culture parameters can all induce systematic shifts in organoid size distribution, lineage proportions, cell type composition, and maturation trajectories. This in turn undermines batch-to-batch consistency and the predictability of functional endpoints [137]. Therefore, for transplantation and translational applications, organoids require production processes organized through quality systems. Secondly, organoid transplantation still carries a non-negligible potential risk of tumorigenicity. Any residual undifferentiated or poorly differentiated cells may form teratomas or abnormal proliferations in vivo. Consequently, the removal of stem cell traces and residual detection must be established as stringent QC measures prior to transplantation. Recent multicenter studies have validated the use of highly sensitive molecular assays such as ddPCR for controlling the in vivo tumorigenic risk of residual pluripotent cells in cell therapy products, providing methodological foundations for cross-laboratory consistency assessment [138]. Concurrently, label-free, high-throughput process-based elimination strategies have emerged for transplant candidate cells such as iPSC-derived spinal cord progenitor cells, aimed at reducing the probability of tumorigenicity arising from residual undifferentiated cells [139]. Furthermore, concerning in vitro tumorigenicity assessment, international multicenter studies on the validation and optimization of highly efficient culture-based detection systems have provided a more actionable pathway for screening residual undifferentiated cells using standardized in vitro assays prior to transplantation [140,141]. The corresponding consensus discussions also emphasized that both carcinogenic risk assessment and release testing should be integrated throughout the entire process, rather than relying solely on end-of-line spot checks [142]. Thirdly, immunocompatibility represents another critical bottleneck determining organoid translatability. Autologous iPSCs theoretically reduce rejection responses, yet their preparation involves lengthy cycles, high costs, and challenges in matching acute/subacute windows. Allogeneic sources typically necessitate immunosuppression or employ cell banks and genetic engineering strategies to mitigate immunogenicity. Recent studies evaluating iPSC haplobank coverage based on global HLA data support the use of HLA-identical cell banks to enhance population compatibility and accessibility [143]. A systematic review of clinical-grade iPSC-derived products further indicates that immunocompatibility strategies must be co-designed with manufacturing, QC, long-term safety, and regulatory frameworks to avoid introducing new safety and quality risks while reducing immune recognition [144].

### OEVs: Translational challenges and countermeasures

When OEVs are directed toward central nervous system indications such as SCI, they are initially constrained by yield bottlenecks in deliverable doses. Under 3D culture conditions, the yield from a single batch of OEVs is often insufficient to support multiple in vivo administrations. Although organoids possess sustained secretory potential, OEV output may vary with organoid-specific upstream variables, including developmental stage, regional identity, cellular composition, matrix conditions, oxygen and nutrient gradients, shear stress, and other culture-associated stresses. These variables may alter both secretion rate and cargo composition and should therefore be treated as upstream process parameters rather than uncontrolled culture variation. A more translatable OEV manufacturing strategy would require controllable, high-density, and perfusable organoid culture integrated with closed downstream processing. Evidence supporting hollow-fiber or perfusion bioreactors, automated bioreactors, and continuous production currently derives predominantly from conventional cell-derived EV systems, where these approaches can improve productivity per unit volume and reduce batch-to-batch variation [145–147]. These studies therefore provide transferable manufacturing principles for OEV scale-up rather than direct validation of neural OEV production. A second major challenge is the lack of standardization, which limits comparability across studies and complicates product release. Current work remains highly heterogeneous in separation and purification methods, dose metrics, administration routes, and dosing frequency, making it difficult to compare efficacy across studies or establish stable dose–potency relationships. Dose definition also requires explicit normalization. Particle number provides a practical measure of administered vesicle abundance but does not by itself reflect cargo content or biological activity, whereas total protein may be affected by co-isolated nonvesicular proteins. A source-organoid equivalent can help relate OEV output to upstream production, but its interpretation depends on organoid size, developmental stage, cellular composition, and culture duration and therefore cannot substitute for a final-product dose metric. Accordingly, early OEV studies should report more than one normalization parameter where feasible, for example, particle number together with protein content and a defined source-organoid or culture-input equivalent, and progressively link these measures to functional potency and in vivo exposure. One practical way forward is to define identity, purity, and potency as critical quality attributes and support them with measurable indicators for product release and batch comparison. For example, consistency across OEV batches could be evaluated using multi-marker profiling to determine whether different preparations meet predefined comparability criteria [148]. General regulatory and quality guidance for exosome- and EV-based therapeutic products emphasizes source control, residues and impurities, stability, potency testing, and nonclinical safety evaluation within an integrated quality system [149,150]. These principles provide a relevant regulatory reference for OEV development, but they should not be interpreted as an established OEV-specific regulatory framework. Targeted delivery remains another key bottleneck because it determines the extent of local exposure at the lesion site. Native OEVs show broad in vivo distribution and are susceptible to clearance by the mononuclear phagocyte system, which reduces their effective accumulation in target tissue. Engineered targeting peptides or membrane proteins may improve lesion-site enrichment, but their development should be accompanied by rigorous assessment of biodistribution, safety—including immunological and hemostatic responses—and manufacturing consistency, to avoid introducing new sources of variability [151].

### Regulatory science framework and exploration of industrialization pathways

The regulatory and industrial challenges of OEVs arise largely from their dual nature. At present, however, a dedicated regulatory framework specifically established for organoid-derived EV products has not yet been defined; therefore, the principles discussed here are extrapolated from organoid manufacturing guidance and broader exosome/EV regulatory frameworks. On the one hand, upstream organoids function as biological production sources and therefore require controls similar to those applied to starting materials in cell therapy. On the other hand, downstream OEV products are developed as formulatable bioactive agents and must meet CMC and release requirements relevant to biologics or nanomedicines. Accordingly, regulatory evaluation should be based on risk stratification and the primary mechanism of action, with organoids, OEVs, and formulation or carrier systems assessed as separable but connected modules. For the organoid component, priority should be given to standardized culture conditions and consistent production criteria, including control of developmental stage, regional identity, cellular composition, matrix conditions, oxygen and nutrient gradients, and culture-associated stress, together with key quality attributes and release thresholds that reduce the risk of starting-material variation affecting OEV cargo and function [152]. For OEV products, an evidence framework compatible with drug development should be established across production, isolation, characterization, potency testing, and safety assessment. This framework also needs to remain aligned with regional regulatory practices for exosome- and EV-based therapies so as to avoid gaps between research-stage feasibility and clinical-grade regulatory readiness [153,154].

For industrialization, scale-up alone is not sufficient; what matters equally is whether product comparability and release can still be maintained after expansion. Recent discussion on EV manufacturing has increasingly shifted from simple volume expansion to an integrated strategy that combines upstream perfusion bioreactors, downstream closed continuous purification, and comparability control. On the manufacturing side, bioreactor scale-up is now being discussed in relation to the need to connect critical process parameters—such as perfusion rate, shear stress, nutrient supply, and harvest window—with key quality attributes, including particle size, marker profile, impurity profile, and functional readouts. This helps expand production while limiting batch drift and supporting cGMP-compatible manufacturing [155]. Because these manufacturing studies are based primarily on conventional EV systems, the corresponding process parameters should be regarded as transferable starting points for OEV manufacturing rather than OEV-specific validated specifications. At the same time, summaries of clinical studies have repeatedly highlighted substantial heterogeneity among current EV products in isolation methods, quantification criteria, and dose description, which makes cross-study efficacy comparison and trial-to-trial extrapolation difficult. For this reason, development needs to place greater emphasis on reproducibility, measurability, and traceability, with a clearer framework for dose definition, potency evaluation, and release criteria [156]. For OEVs, this logic must extend further upstream to organoid production. It is particularly important to establish a traceable link between organoid developmental stage or tissue identity and the resulting OEV cargo profiles and potency endpoints so that platform-related variation can be monitored and controlled rather than treated as uncontrollable noise [152,155].

A more practical way to support OEV industrialization is to encourage early sharing of a basic set of comparable data among academia, industry, and regulatory stakeholders. Publicly accessible frameworks for characterization and potency reporting, together with reproducible process descriptions and cross-platform comparison studies, may help move OEVs from proof-of-concept materials toward registrable engineered biologics. Guidance reviews for clinical translation also indicate that safety (e.g., risks associated with impurities and residues), consistency (batch-to-batch comparability), and clinical interpretability (the dose-exposure-potency chain) should be prioritized as primary constraints to inform reverse-engineered research and development design. This approach shortens the iteration cycle from laboratory to IND/clinical trials [153,154,156].

## Conclusions and Outlook

Neural organoids and OEVs provide complementary opportunities for SCI repair, but several key questions remain unresolved. Most importantly, it remains unclear whether neural OEVs possess reproducible source- or developmental-state-associated features that translate into distinct functional advantages in SCI. The relationships among source-organoid identity, OEV cargo, biological potency, and therapeutic outcome also remain insufficiently defined. In addition, the optimal timing, dose, and delivery route for OEV intervention, as well as the conditions under which OEVs provide added value to organoid transplantation, require direct comparative validation.

Future research should therefore prioritize prospective studies that establish source–cargo–function relationships, define mechanism-linked potency assays, and test stage- and exposure-matched treatment strategies using appropriate EV and combination controls. Equally important will be the development of reproducible manufacturing and QC frameworks that link source-organoid critical quality attributes with final OEV product attributes, establish interpretable dose-normalization metrics, and integrate mechanism-linked potency assays with product characterization, safety evaluation, and batch-release criteria. Such frameworks should distinguish general EV characterization from functional potency assessment and clarify how upstream variation in organoid state is propagated into downstream OEV quality and activity. Addressing these priorities will determine whether neural organoids and OEVs can progress from promising experimental platforms toward reproducible and clinically interpretable regenerative strategies for SCI.

## Data Availability

Data sharing is not applicable to this article as no datasets were generated or analyzed during the current study.

## References

[1] World Health Organization. Spinal cord injury. 16 Apr 2024. [cited 15 Feb 2026] https://www.who.int/zh/news-room/fact-sheets/detail/spinal-cord-injury

[2] GBD Spinal Cord Injuries Collaborators. Global, regional, and national burden of spinal cord injury, 1990-2019: A systematic analysis for the Global Burden of Disease Study 2019. Lancet Neurol. 2023;22(11):1026–1047. 10.1016/S1474-4422(23)00287-9 PMC10584692 37863591

[3] Fan B, Wei Z, Feng S. Progression in translational research on spinal cord injury based on microenvironment imbalance. Bone Res. 2022;10:35. 10.1038/s41413-022-00199-9 PMC8993811 35396505

[4] Hu X, Xu W, Ren Y, Wang Z, He X, Huang R, Ma B, Zhao J, Zhu R, Cheng L. Spinal cord injury: Molecular mechanisms and therapeutic interventions. Signal Transduct Target Ther. 2023;8:245. 10.1038/s41392-023-01477-6 PMC10291001 37357239

[5] Andrews MG, Kriegstein AR. Challenges of organoid research. Annu Rev Neurosci. 2022;45:23–39. 10.1146/annurev-neuro-111020-090812 PMC10559943 34985918

[6] Lee JH, Shin H, Shaker MR, Kim HJ, Park SH, Kim JH, Lee N, Kang M, Cho S, Kwak TH, et al. Production of human spinal-cord organoids recapitulating neural-tube morphogenesis. Nat Biomed Eng. 2022;6(4):435–448. 10.1038/s41551-022-00868-4 35347276

[7] Revah O, Gore F, Kelley KW, Andersen J, Sakai N, Chen X, et al. Maturation and circuit integration of transplanted human cortical organoids. Nature. 2022;610:319–326. 10.1038/s41586-022-05277-w PMC9556304 36224417

[8] Xu B, Liu D, Liu W, Long G, Liu W, Wu Y, He X, Shen Y, Jiang P, Yin M, et al. Engineered human spinal cord-like tissues with dorsal and ventral neuronal progenitors for spinal cord injury repair in rats and monkeys. Bioact Mater. 2023;27:125–137. 10.1016/j.bioactmat.2023.03.015 PMC10090126 37064803

[9] Lai BQ, Wu RJ, Han WT, Bai YR, Liu JL, Yu HY, Yang SB, Wang LJ, Ren JL, Ding Y, et al. Tail nerve electrical stimulation promoted the efficiency of transplanted spinal cord-like tissue as a neuronal relay to repair the motor function of rats with transected spinal cord injury. Biomaterials. 2023;297: Article 122103. 10.1016/j.biomaterials.2023.122103 37028111

[10] Sun Z, Chen Z, Yin M, Wu X, Guo B, Cheng X, Quan R, Sun Y, Zhang Q, Fan Y, et al. Harnessing developmental dynamics of spinal cord extracellular matrix improves regenerative potential of spinal cord organoids. Cell Stem Cell. 2024;31(5):772–787.e11. 10.1016/j.stem.2024.03.007 38565140

[11] Welsh JA, Goberdhan DCI, O’Driscoll L, Buzas EI, Blenkiron C, Bussolati B, Cai H, Di Vizio D, Driedonks TAP, Erdbrügger U, et al. Minimal information for studies of extracellular vesicles (MISEV2023): From basic to advanced approaches. J Extracell Vesicles. 2024;13: Article e12404. 10.1002/jev2.12404 PMC10850029 38326288

[12] Rong Y, Liu W, Wang J, Fan J, Luo Y, Li L, Kong F, Chen J, Tang P, Cai W. Neural stem cell-derived small extracellular vesicles attenuate apoptosis and neuroinflammation after traumatic spinal cord injury by activating autophagy. Cell Death Dis. 2019;10:340. 10.1038/s41419-019-1571-8 PMC6472377 31000697

[13] Fang A, Wang Y, Guan N, Zuo Y, Lin L, Guo B, Mo A, Wu Y, Lin X, Cai W, et al. Porous microneedle patch with sustained delivery of extracellular vesicles mitigates severe spinal cord injury. Nat Commun. 2023;14:4011. 10.1038/s41467-023-39745-2 PMC10328956 37419902

[14] Chen G, Tong K, Li S, Huang Z, Liu S, Zhu H, Zhong Y, Zhou Z, Jiao G, Wei F, et al. Extracellular vesicles released by transforming growth factor-beta 1-preconditional mesenchymal stem cells promote recovery in mice with spinal cord injury. Bioact Mater. 2024;35:135–149. 10.1016/j.bioactmat.2024.01.013 PMC10837068 38312519

[15] Peng W, Zhang W, Cui W, Chen W, Zhuang Y, Chu R, Tan J, Xue J, Yan Y, Yin G, et al. Engineered small extracellular vesicles for targeted delivery of perlecan to stabilise the blood-spinal cord barrier after spinal cord injury. Clin Transl Med. 2025;15(6): Article e70381. 10.1002/ctm2.70381 PMC12179339 40538064

[16] Gao H, Zeng Y, Huang X, A L, Liang Q, Xie J, Lin X, Gong J, Fan X, Zou T, et al. Extracellular vesicles from organoid-derived human retinal progenitor cells prevent lipid overload-induced retinal pigment epithelium injury by regulating fatty acid metabolism. J Extracell Vesicles. 2024;13(1): Article e12401. 10.1002/jev2.12401 PMC10752800 38151470

[17] Zhang M, Han X, Yan L, Fu Y, Kou H, Shang C, Wang J, Liu H, Jiang C, Wang J, et al. Inflammatory response in traumatic brain and spinal cord injury: The role of XCL1-XCR1 axis and T cells. CNS Neurosci Ther. 2024;30(6): Article e14781. 10.1111/cns.14781 PMC11183917 38887195

[18] Theus MH. Neuroinflammation and acquired traumatic CNS injury: A mini review. Front Neurol. 2024;15: Article 1334847. 10.3389/fneur.2024.1334847 PMC10915049 38450073

[19] Jiang Z, Zhang C, Zhao Z, Ning B. Blood-spinal cord barrier disruption after spinal cord injury: A time-dependent mechanistic review. Front Cell Neurosci. 2026;20: Article 1805529. 10.3389/fncel.2026.1805529 PMC13043419 41939131

[20] Nascimento D, Ferreira A, Cruz CD. Immune activation following spinal cord injury: A review focused on inflammatory changes in the spinal cord. Int J Mol Sci. 2025;26(19):9624. 10.3390/ijms26199624 PMC12525225 41096889

[21] Zeng WC, Zeng FJ. Regulatory effect of inflammatory mediators in spinal cord injury. Front Immunol. 2025;16: Article 1619337. 10.3389/fimmu.2025.1619337 PMC12417158 40934001

[22] Feng Z, Min L, Liang L, Chen B, Chen H, Zhou Y, Deng W, Liu H, Hou J. Neutrophil extracellular traps exacerbate secondary injury via promoting neuroinflammation and blood-spinal cord barrier disruption in spinal cord injury. Front Immunol. 2021;12: Article 698249. 10.3389/fimmu.2021.698249 PMC8385494 34456910

[23] Pang QM, Chen SY, Xu QJ, Fu SP, Yang YC, Zou WH, Zhang T. Neuroinflammation and scarring after spinal cord injury: Therapeutic roles of MSCs on inflammation and glial scar. Front Immunol. 2021;12: Article 751021. 10.3389/fimmu.2021.751021 PMC8674561 34925326

[24] Shafqat A, Albalkhi I, Magableh HM, Saleh T, Alkattan K, Yaqinuddin A. Tackling the glial scar in spinal cord regeneration: New discoveries and future directions. Front Cell Neurosci. 2023;17: Article 1180825. 10.3389/fncel.2023.1180825 PMC10244598 37293626

[25] Tran AP, Warren PM, Silver J. New insights into glial scar formation after spinal cord injury. Cell Tissue Res. 2022;387(3):319–336. 10.1007/s00441-021-03477-w PMC8975767 34076775

[26] Yari D, Saberi A, Salmasi Z, Ghoreishi SA, Etemad L, Movaffagh J, Pasdar A. Recent advances in the treatment of spinal cord injury. Arch Bone Jt Surg. 2024;12(6):380–399. 10.22038/ABJS.2023.73944.3424 PMC11195032 38919744

[27] Okano Y, Kase Y, Suematsu Y, Nakamura M, Okano H. Chronological transitions of hepatocyte growth factor treatment effects in spinal cord injury tissue. Inflamm Regen. 2024;44:10. 10.1186/s41232-024-00322-9 PMC10935783 38475915

[28] Zhou G, Li R, Sheng S, Huang J, Zhou F, Wei Y, Liu H, Su J. Organoids and organoid extracellular vesicles-based disease treatment strategies. J Nanobiotechnol. 2024;22:679. 10.1186/s12951-024-02917-3 PMC11542470 39506799

[29] Xue W, Li B, Liu H, Xiao Y, Li B, Ren L, Li H, Shao Z. Generation of dorsoventral human spinal cord organoids via functionalizing composite scaffold for drug testing. iScience. 2023;26(1): Article 105898. 10.1016/j.isci.2022.105898 PMC9840144 36647382

[30] Seo K, Cho S, Shin H, Shin A, Lee JH, Kim JH, Lee B, Jang H, Kim Y, Cho HM, et al. Symmetry breaking of human pluripotent stem cells (hPSCs) in micropattern generates a polarized spinal cord-like organoid (pSCO) with dorsoventral organization. Adv Sci. 2023;10(20): Article e2301787. 10.1002/advs.202301787 PMC10369253 37170679

[31] Guo R, Chen Y, Zhang J, Zhou Z, Feng B, Du X, Liu X, Ma J, Cui H. Neural differentiation and spinal cord organoid generation from induced pluripotent stem cells (iPSCs) for ALS modelling and inflammatory screening. Mol Neurobiol. 2024;61:4732–4749. 10.1007/s12035-023-03836-4 38127186

[32] McDonald M, Sebinger D, Brauns L, Gonzalez-Cano L, Menuchin-Lasowski Y, Mierzejewski M, Psathaki OE, Stumpf A, Wickham J, Rauen T, et al. A mesh microelectrode array for non-invasive electrophysiology within neural organoids. Biosens Bioelectron. 2023;228: Article 115223. 10.1016/j.bios.2023.115223 36931193

[33] Iyer NR, Ashton RS. Bioengineering the human spinal cord. Front Cell Dev Biol. 2022;10: Article 942742. 10.3389/fcell.2022.942742 PMC9458954 36092702

[34] Zhou G, Pang S, Li Y, Gao J. Progress in the generation of spinal cord organoids over the past decade and future perspectives. Neural Regen Res. 2024;19:1013–1019. 10.4103/1673-5374.385280 PMC10749595 37862203

[35] Rabeling A, van der Hoven A, Andersen N, Goolam M. Neural tube organoids: A novel system to study developmental timing. Stem Cell Rev Rep. 2024;20(8):2045–2061. 10.1007/s12015-024-10785-5 PMC11554929 39230820

[36] Luo T, Liu C, Cheng T, Zhao GQ, Huang Y, Luan JY, Guo J, Liu X, Wang YF, Dong Y, et al. Establishing dorsal-ventral patterning in human neural tube organoids with synthetic organizers. Cell Stem Cell. 2025;32(7):1071–1086.e8. 10.1016/j.stem.2025.04.011 40373768

[37] Elkouris M, Balaskas N, Poulou M, Politis PK, Panayiotou E, Malas S, et al. Sox1 maintains the undifferentiated state of cortical neural progenitor cells via the suppression of Prox1-mediated cell cycle exit and neurogenesis. Stem Cells. 2011;29(1):89–98. 10.1002/stem.554 21280160

[38] Karnavas T, Mandalos N, Malas S, Remboutsika E. SoxB, cell cycle and neurogenesis. Front Physiol. 2013;4:298. 10.3389/fphys.2013.00298 PMC3797971 24146653

[39] Dhar A, Moinuddin FM, Zamanian CA, Sharar AD, Dominari A, Graepel S, et al. SOX genes in spinal cord injury: Redefining neural stem cell regeneration strategies. Mol Neurobiol. 2025;62:9772–9781. 10.1007/s12035-025-04882-w 40156684

[40] Xue X, Kim YS, Ponce-Arias AI, O’Laughlin R, Yan RZ, Kobayashi N, Tshuva RY, Tsai YH, Sun S, Zheng Y, et al. A patterned human neural tube model using microfluidic gradients. Nature. 2024;628(8007):391–399. 10.1038/s41586-024-07204-7 PMC11006583 38408487

[41] Phouphetlinthong O, Partiot E, Bernou C, Sebban A, Gaudin R, Charlot B. Protruding cantilever microelectrode array to monitor the inner electrical activity of cerebral organoids. Lab Chip. 2023;23(16):3603–3614. 10.1039/d3lc00294b 37489118

[42] Ao Z, Cai H, Wu Z, Krzesniak J, Tian C, Lai YY, et al. Human spinal organoid-on-a-chip to model nociceptive circuitry for pain therapeutics discovery. Anal Chem. 2022;94(2):1365–1372. 10.1021/acs.analchem.1c04641 PMC11483356 34928595

[43] Chooi WH, Wu Y, Ng SY. Defined hydrogels for spinal cord organoids: Challenges and potential applications. Neural Regen Res. 2024;19(11):2329–2330. 10.4103/NRR.NRR-D-23-01665 PMC11090425 38526259

[44] Chooi WH, Ng CY, Ow V, Harley J, Ng W, Hor JH, Low KE, Malleret B, Xue K, Ng SY. Defined alginate hydrogels support spinal cord organoid derivation, maturation, and modeling of spinal cord diseases. Adv Healthc Mater. 2023;12(9): Article e2202342. 10.1002/adhm.202202342 36502337

[45] Takata N, Li Z, Metlushko A, Chen F, Sather NA, Lin X, Schipma MJ, Carballo-Molina OA, Jamroz C, Strong ME, et al. Injury and therapy in a human spinal cord organoid. Nat Biomed Eng. 2026;10(2):234–246. 10.1038/s41551-025-01606-2 41673139

[46] Liu H, Su J. Organoid extracellular vesicle-based therapeutic strategies for bone therapy. Biomater Transl. 2023;4(4):199–212. 10.12336/biomatertransl.2023.04.002 PMC10817793 38282702

[47] Abdal Dayem A, Kwak Y, Jeun H, Cho SG. Recent insights into organoid-derived extracellular vesicles and their biomedical applications. J Pers Med. 2025;15(10):492. 10.3390/jpm15100492 PMC12564934 41149854

[48] Ene J, Muok L, Gonzalez V, Sanchez N, Nathani A, Syed F, et al. Biomanufacturing and curcumin-loading of human choroid plexus organoid-derived extracellular vesicles from a vertical-wheel bioreactor to alleviate neuro-inflammation. Biomedicines. 2025;13(5):1069. 10.3390/biomedicines13051069 PMC12109122 40426897

[49] Liu C, Ene J, Lu W, Syed F, Sun L, Raulin AC, Ren Y, Wang X, Kanekiyo T, Li Y. Immuno-regulation of brain region-specific organoids containing isogenic microglia-like cells. Adv Healthc Mater. 2026;15(10): Article e03579. 10.1002/adhm.202503579 PMC12967306 41408986

[50] Wang Z, Zhou X, Kong Q, He H, Sun J, Qiu W, Zhang L, Yang M. Extracellular vesicle preparation and analysis: A state-of-the-art review. Adv Sci. 2024;11: Article e2401069. 10.1002/advs.202401069 PMC11321646 38874129

[51] Abdollahi S. Extracellular vesicles from organoids and 3D culture systems. Biotechnol Bioeng. 2021;118(3):1029–1049. 10.1002/bit.27606 33085083

[52] Schuster M, Braun FK, Chiang DM, Ludwig C, Meng C, Grätz C, Kirchner B, Proescholdt M, Hau P, Steinlein OK, et al. Extracellular vesicles secreted by 3D tumor organoids are enriched for immune regulatory signaling biomolecules compared to conventional 2D glioblastoma cell systems. Front Immunol. 2024;15: Article 1388769. 10.3389/fimmu.2024.1388769 PMC11079215 38726003

[53] Bahram Sangani N, Koetsier J, Gomes AR, Diogo MM, Fernandes TG, Bouwman FG, Mariman ECM, Ghazvini M, Gribnau J, Curfs LMG, et al. Involvement of extracellular vesicle microRNA clusters in developing healthy and Rett syndrome brain organoids. Cell Mol Life Sci. 2024;81:410. 10.1007/s00018-024-05409-7 PMC11416455 39305343

[54] Stankovic I, Smit P, Cross J, Rai A, Wolujewicz P, Greening D, Colak D. Extracellular vesicle profiling reveals novel autism signatures in patient-derived forebrain organoids. Transl Psychiatry. 2025;15(1):393. 10.1038/s41398-025-03607-w PMC12514172 41073401

[55] Liu C, Chen X, Ene J, Esmonde C, Kanekiyo T, Zeng C, Sun L, Li Y. Engineering extracellular vesicles secreted by human brain organoids with different regional identity. ACS Appl Mater Interfaces. 2025;17(10):15145–15162. 10.1021/acsami.4c22692 PMC12505154 40030083

[56] Saint-Pol J, Culot M. Minimum information for studies of extracellular vesicles (MISEV) as toolbox for rigorous, reproducible and homogeneous studies on extracellular vesicles. Toxicol In Vitro. 2025;106: Article 106049. 10.1016/j.tiv.2025.106049 40074066

[57] Visan KS, Lobb RJ, Ham S, Lima LG, Palma C, Edna CPZ, Wu LY, Gowda H, Datta KK, Hartel G, et al. Comparative analysis of tangential flow filtration and ultracentrifugation, both combined with subsequent size exclusion chromatography, for the isolation of small extracellular vesicles. J Extracell Vesicles. 2022;11: Article e12266. 10.1002/jev2.12266 PMC9486818 36124834

[58] Bonner SE, van de Wakker SI, Phillips W, Willms E, Sluijter JPG, Hill AF, Vader P. Scalable purification of extracellular vesicles with high yield and purity using multimodal flowthrough chromatography. J Extracell Biol. 2024;3: Article e138. 10.1002/jex2.138 PMC11080796 38939900

[59] Suresh PS, Zhang Q. Comprehensive comparison of methods for isolation of extracellular vesicles from human plasma. J Proteome Res. 2025;24(6):2956–2967. 10.1021/acs.jproteome.5c00149 PMC12150312 40356199

[60] Nieland L, Mahjoum S, Grandell E, Breyne K, Breakefield XO. Engineered EVs designed to target diseases of the CNS. J Control Release. 2023;356:493–506. 10.1016/j.jconrel.2023.03.009 PMC10226535 36907561

[61] Upadhya D, Shetty AK. MISEV2023 provides an updated and key reference for researchers studying the basic biology and applications of extracellular vesicles. Stem Cells Transl Med. 2024;13:848–850. 10.1093/stcltm/szae052 PMC11386207 39028333

[62] Rene CA, Parks RJ. Bioengineering extracellular vesicle cargo for optimal therapeutic efficiency. Mol Ther Methods Clin Dev. 2024;32: Article 101259. 10.1016/j.omtm.2024.101259 PMC11103572 38770107

[63] Mohammadi AH, Ghazvinian Z, Bagheri F, Harada M, Baghaei K. Modification of extracellular vesicle surfaces: An approach for targeted drug delivery. BioDrugs. 2023;37:353–374. 10.1007/s40259-023-00595-5 37093521

[64] Mediratta K, Diab MD, Han P, Hu H, Wang L. Emerging strategies for cargo loading and engineering of extracellular vesicles for breast cancer treatment. Nanomaterials. 2025;15(18):1418. 10.3390/nano15181418 PMC12472437 41003053

[65] Li C, Wu Z, Zhou L, Shao J, Hu X, Xu W, Ren Y, Zhu X, Ge W, Zhang K, et al. Temporal and spatial cellular and molecular pathological alterations with single-cell resolution in the adult spinal cord after injury. Signal Transduct Target Ther. 2022;7(1):65. 10.1038/s41392-022-00885-4 PMC8888618 35232960

[66] Milich LM, Choi JS, Ryan C, Cerqueira SR, Benavides S, Yahn SL, Tsoulfas P, Lee JK, et al. Single-cell analysis of the cellular heterogeneity and interactions in the injured mouse spinal cord. J Exp Med. 2021;218: Article e20210040. 10.1084/jem.20210040 PMC8212781 34132743

[67] Li Y, Gu Y, Wang Z, Wang Y, Jiang S, Wang K, Zheng Y, Feng R, Yang M. GelMA hydrogel encapsulating iPSC-derived human spinal cord organoids enhances neural regeneration and restores motor function in rat spinal cord injury. J Biomed Mater Res A. 2025;113(10): Article e38001. 10.1002/jbm.a.38001 41051068

[68] Wang Z, Zhao H, Tang X, Meng T, Khutsishvili D, Xu B, Ma S. CNS organoid surpasses cell-laden microgel assembly to promote spinal cord injury repair. Research. 2022;2022: Article 9832128. 10.34133/2022/9832128 PMC9394056 36061824

[69] Ma L, Zhang Z, Mu Y, Liu B, Jin M, Ma C, Zhou H, Zhang Y, Wang DA. Generation of engineered human spinal cord organoid in channel-patterned collagen and transplantation for spinal cord regeneration. Biomaterials. 2026;327: Article 123753. 10.1016/j.biomaterials.2025.123753 41061610

[70] Li E, Yan R, Yan K, Zhang R, Zhang Q, Zou P, Wang Y, Qiao Y, Li Y, Ma B, et al. Single-cell RNA sequencing reveals the role of immune-related autophagy in spinal cord injury in rats. Front Immunol. 2022;13: Article 987344. 10.3389/fimmu.2022.987344 PMC9535363 36211348

[71] Jin LY, Li J, Wang KF, Xia WW, Zhu ZQ, Wang CR, Li XF, Liu HY. Blood-spinal cord barrier in spinal cord injury: A review. J Neurotrauma. 2021;38(9):1203–1224. 10.1089/neu.2020.7413 33292072

[72] Zhou R, Li J, Chen Z, Wang R, Shen Y, Zhang R, Zhou F, Zhang Y. Pathological hemodynamic changes and leukocyte transmigration disrupt the blood-spinal cord barrier after spinal cord injury. J Neuroinflammation. 2023;20(1):118. 10.1186/s12974-023-02787-w PMC10200062 37210532

[73] Xie Y, Luo Z, Peng W, Liu Y, Yuan F, Xu J, Sun Y, Lu H, Wu T, Jiang L, et al. Inhibition of UTX/KDM6A improves recovery of spinal cord injury by attenuating BSCB permeability and macrophage infiltration through the MLCK/p-MLC pathway. J Neuroinflammation. 2023;20(1):259. 10.1186/s12974-023-02936-1 PMC10638785 37951955

[74] Zhu S, Chen M, Ying Y, Wu Q, Huang Z, Ni W, Wang X, Xu H, Bennett S, Xiao J, et al. Versatile subtypes of pericytes and their roles in spinal cord injury repair, bone development and repair. Bone Res. 2022;10(1):30. 10.1038/s41413-022-00203-2 PMC8927336 35296645

[75] Park CS, Lee JY, Yune TY. Imatinib prevents blood-spinal cord barrier disruption by inhibiting PDGFR-mediated JMJD3 expression and activation after spinal cord injury. Fluids Barriers CNS. 2025;22:76. 10.1186/s12987-025-00690-5 PMC12269138 40671131

[76] Dithmer S, Blasig IE, Fraser PA, Qin Z, Haseloff RF. The basic requirement of tight junction proteins in blood-brain barrier function and their role in pathologies. Int J Mol Sci. 2024;25(16):9164. 10.3390/ijms25115601 PMC11172262 38891789

[77] Nie X, Liu Y, Yuan T, Yu T, Yun Z, Xue W, Yu T, An J, Dai A, Wu K, et al. Platelet-rich plasma-derived exosomes promote blood-spinal cord barrier repair and attenuate neuroinflammation after spinal cord injury. J Nanobiotechnol. 2024;22:456. 10.1186/s12951-024-02737-5 PMC11290287 39085856

[78] Kumamaru H, Lu P, Rosenzweig ES, Kadoya K, Tuszynski MH. Regenerating corticospinal axons innervate phenotypically appropriate neurons within neural stem cell grafts. Cell Rep. 2019;26(9):2329–2339.e4. 10.1016/j.celrep.2019.01.099 PMC6487864 30811984

[79] Zhu Y, Huang R, Yu L, Liu Z, Liu Y, Fan W, Lin G, Wang Z, He X, Xu X, et al. Engineered thoracic spinal cord organoids for transplantation after spinal cord injury. Nat Biomed Eng. 2025. 10.1038/s41551-025-01549-8 PMC13672100 41136603

[80] Hu N, Shi JX, Chen C, Xu HH, Chang ZH, Hu PF, Guo D, Zhang XW, Shao WW, Fan X, et al. Constructing organoid-brain-computer interfaces for neurofunctional repair after brain injury. Nat Commun. 2024;15(1). 10.1038/s41467-024-53858-2 PMC11541701 39505863

[81] Jgamadze D, Lim JT, Zhang Z, Harary PM, Germi J, Mensah-Brown K, Adam CD, Mirzakhalili E, Singh S, Gu JB, et al. Structural and functional integration of human forebrain organoids with the injured adult rat visual system. Cell Stem Cell. 2023;30(2):137–152.e7. 10.1016/j.stem.2023.01.004 PMC9926224 36736289

[82] Wilson MN, Thunemann M, Liu X, Lu Y, Puppo F, Adams JW, et al. Multimodal monitoring of human cortical organoids implanted in mice reveal functional connection with visual cortex. Nat Commun. 2022;13(1):7945. 10.1038/s41467-022-35536-3 PMC9792589 36572698

[83] Ceto S, Sekiguchi KJ, Takashima Y, Nimmerjahn A, Tuszynski MH. Neural stem cell grafts form extensive synaptic networks that integrate with host circuits after spinal cord injury. Cell Stem Cell. 2020;27(3):430–440.e5. 10.1016/j.stem.2020.07.007 PMC7484050 32758426

[84] Cao SY, Yang D, Huang ZQ, Lin YH, Wu HY, Chang L, Luo CX, Xu Y, Liu Y, Zhu DY. Cerebral organoids transplantation repairs infarcted cortex and restores impaired function after stroke. npj Regener Med. 2023;8:27. 10.1038/s41536-023-00301-7 PMC10229586 37253754

[85] Hosseini SM, Alizadeh A, Shahsavani N, Chopek J, Ahlfors JE, Karimi-Abdolrezaee S. Suppressing CSPG/LAR/PTPsigma axis facilitates neuronal replacement and synaptogenesis by human neural precursor grafts and improves recovery after spinal cord injury. J Neurosci. 2022;42(15):3096–3121. 10.1523/JNEUROSCI.2177-21.2022 PMC8994547 35256527

[86] Zeldich E, Rajkumar S. Identity and maturity of iPSC-derived oligodendrocytes in 2D and organoid systems. Cells. 2024;13(8):674. 10.3390/cells13080674 PMC11049552 38667289

[87] James OG, Selvaraj BT, Magnani D, Burr K, Connick P, Barton SK, Vasistha NA, Hampton DW, Story D, Smigiel R, et al. iPSC-derived myelinoids to study myelin biology of humans. Dev Cell. 2021;56(9):1346–1358 e6. 10.1016/j.devcel.2021.04.006 PMC8098746 33945785

[88] Feng L, Chao J, Zhang M, Pacquing E, Hu W, Shi Y. Developing a human iPSC-derived three-dimensional myelin spheroid platform for modeling myelin diseases. iScience. 2023;26: Article 108037. 10.1016/j.isci.2023.108037 PMC10589867 37867939

[89] Marangon D, Castro ESJH, Cerrato V, Boda E, Lecca D. Oligodendrocyte progenitors in glial scar: A bet on remyelination. Cells. 2024;13(12): Article 1024. 10.3390/cells13121024 PMC11202012 38920654

[90] Li Q, Liu S, Zheng T, Li M, Qi B, Zhou L, Liu B, Ma D, Zhao C, Chen Z. Grafted human-induced pluripotent stem cells-derived oligodendrocyte progenitor cells combined with human umbilical vein endothelial cells contribute to functional recovery following spinal cord injury. Stem Cell Res Ther. 2024;15:35. 10.1186/s13287-024-03651-1 PMC10848469 38321505

[91] Patil N, Walsh P, Carrabre K, Holmberg EG, Lavoie N, Dutton JR, Parr AM. Regionally specific human pre-oligodendrocyte progenitor cells produce both oligodendrocytes and neurons after transplantation in a chronically injured spinal cord rat model after glial scar ablation. J Neurotrauma. 2021;38(6):777–788. 10.1089/neu.2020.7009 33107383

[92] Chen K, Yu W, Zheng G, Xu Z, Yang C, Wang Y, Wang X, Zhang J, Li Y, Liu S, et al. Biomaterial-based regenerative therapeutic strategies for spinal cord injury. NPG Asia Mater. 2024;16(1):5.

[93] Li C, Kuss M, Kong Y, Nie F, Liu X, Liu B, Dunaevsky A, Fayad P, Duan B, Li X. 3D printed hydrogels with aligned microchannels to guide neural stem cell migration. ACS Biomater Sci Eng. 2021;7(2):690–700. 10.1021/acsbiomaterials.0c01619 33507749

[94] Han G, Lavoie NS, Patil N, Korenfeld OG, Kim H, Esguerra M, Joung D, McAlpine MC, Parr AM. 3D-printed scaffolds promote enhanced spinal organoid formation for use in spinal cord injury. Adv Healthc Mater. 2025;14: Article e04817. 10.1002/adhm.202404817 PMC12447032 40702833

[95] Siddiqui AM, Oswald D, Papamichalopoulos S, Kelly D, Summer P, Polzin M, Hakim J, Schmeichel AM, Chen B, Yaszemski MJ, et al. Defining spatial relationships between spinal cord axons and blood vessels in hydrogel scaffolds. Tissue Eng Part A. 2021;27(11-12):648–664. 10.1089/ten.tea.2020.0316 PMC8219201 33764164

[96] Hong SJ, Bock M, Zhang S, An SB, Han I. Therapeutic transplantation of human central nervous system organoids for neural reconstruction. Int J Mol Sci. 2024;25(15):8540. 10.3390/ijms25158540 PMC11313261 39126108

[97] An JH, Koh H, Ahn Y, Kim J, Han AR, Lee JY, Kim SU, Lee JH. Maintenance of hypoimmunogenic features via regulation of endogenous antigen processing and presentation machinery. Front Bioeng Biotechnol. 2022;10: Article 936584. 10.3389/fbioe.2022.936584 PMC9416868 36032723

[98] Hu X, White K, Olroyd AG, DeJesus R, Dominguez AA, Dowdle WE, Friera AM, Young C, Wells F, Chu EY, et al. Hypoimmune induced pluripotent stem cells survive long term in fully immunocompetent, allogeneic rhesus macaques. Nat Biotechnol. 2024;42:413–423. 10.1038/s41587-023-01784-x PMC10940156 37156915

[99] Caroli A, Varinelli M, Vigani L, Vallerga A, Rizzo P, Rottoli D, Conti S, Craparotta I, Fumagalli S, Remuzzi G, et al. Hypoimmunogenic iPSC-derived hepatic organoids featuring a functional vascular network. Sci Rep. 2026;16: Article 29104. 10.1038/s41598-026-58977-y PMC13586248 42342802

[100] Zhou J, Flores-Bellver M, Pan J, Benito-Martin A, Shi C, Onwumere O, Mighty J, Qian J, Zhong X, Hogue T, et al. Human retinal organoids release extracellular vesicles that regulate gene expression in target human retinal progenitor cells. Sci Rep. 2021;11(1):21128. 10.1038/s41598-021-00542-w PMC8548301 34702879

[101] Forero A, Pipicelli F, Moser S, Baumann N, Grätz C, Gonzalez Pisfil M, Pfaffl MW, Pütz B, Kielkowski P, Cernilogar FM, et al. Extracellular vesicle-mediated trafficking of molecular cues during human brain development. Cell Rep. 2024;43(10): Article 114755. 10.1016/j.celrep.2024.114755 39302835

[102] Herrera Lopez M, Bertone Arolfo M, Remedi M, Gastaldi L, Wilson C, Guendulain GG, Ceschin D, Cardozo Gizzi A, Cáceres A, Moyano AL. Human neural rosettes secrete bioactive extracellular vesicles enriched in neuronal and glial cellular components. Sci Rep. 2025;15:1987. 10.1038/s41598-025-86094-9 PMC11736123 39814837

[103] Ji X, Zhou S, Wang N, Wang J, Wu Y, Duan Y, Ni P, Zhang J, Yu S. Cerebral-organoid-derived exosomes alleviate oxidative stress and promote LMX1A-dependent dopaminergic differentiation. Int J Mol Sci. 2023;24(13): Article 11048. 10.3390/ijms241311048 PMC10341736 37446226

[104] Arthur P, Kandoi S, Sun L, Kalvala A, Kutlehria S, Bhattacharya S, Kulkarni T, Nimma R, Li Y, Lamba DA, et al. Biophysical, molecular and proteomic profiling of human retinal organoid-derived exosomes. Pharm Res. 2023;40(4):801–816. 10.1007/s11095-022-03350-7 PMC10576571 36002615

[105] Saiduzzaman SM, Xu R, Sampad MJN, Hoffman RN, Seiler ST, Brail Q, Yurevych V, Walker ZJ, Wells TN, Ong EM, et al. Single molecule nanopore counting assay targeting small extracellular vesicle cargo for non-invasive monitoring of cerebral organoid development and health. Sci Rep. 2025;15(1):45806. 10.1038/s41598-025-31284-8 PMC12756300 41387532

[106] Silver BB, Fannin R, Gerrish K, Tokar EJ. Characterization of extracellular vesicles and miRNA released by cerebral organoids. Curr Res Toxicol. 2025;8: Article 100229. 10.1016/j.crtox.2025.100229 PMC12133712 40469891

[107] Kong G, Liu J, Wang J, Yu X, Li C, Deng M, Liu M, Wang S, Tang C, Xiong W, et al. Engineered extracellular vesicles modified by Angiopep-2 peptide promote targeted repair of spinal cord injury and brain inflammation. ACS Nano. 2025;19(4):4582–4600. 10.1021/acsnano.4c14675 PMC11803916 39853366

[108] Xiong W, Liu M, Wang J, Liu J, Zheng M, Chan P, Zhu Q, Li C, Kong G, Tang C, et al. Multidimensional engineering of extracellular vesicles for targeted delivery and microglial reprograming in spinal cord injury repair. ACS Nano. 2025;19(35):31551–31571. 10.1021/acsnano.5c08573 PMC12424966 40845835

[109] Ran N, Li W, Zhang R, Lin C, Zhang J, Wei Z, Li Z, Yuan Z, Wang M, Fan B, et al. Autologous exosome facilitates load and target delivery of bioactive peptides to repair spinal cord injury. Bioact Mater. 2023;25:766–782. 10.1016/j.bioactmat.2022.07.002 PMC10086682 37056263

[110] Cao J, Zhang X, Guo J, Wu J, Lin L, Lin X, Mu J, Huang T, Zhu M, Ma L, et al. An engineering-reinforced extracellular vesicle–integrated hydrogel with an ROS-responsive release pattern mitigates spinal cord injury. Sci Adv. 2025;11(14): Article eads3398. 10.1126/sciadv.ads3398 PMC11963969 40173229

[111] Liang X, Niu Z, Galli V, Howe N, Zhao Y, Wiklander OPB, Smith CIE, El Andaloussi S, Nordin JZ, Corso G. Extracellular vesicles engineered to bind albumin demonstrate extended circulation time and lymph node accumulation in mouse models. J Extracell Vesicles. 2022;11: Article e12248. 10.1002/jev2.12248 PMC9314316 35879268

[112] Garcia SG, Sanroque-Muñoz M, Clos-Sansalvador M, Font-Morón M, Monguió-Tortajada M, Borràs FE, Franquesa M. Hollow fiber bioreactor allows sustained production of immortalized mesenchymal stromal cell-derived extracellular vesicles. Extracell Vesicles Circ Nucl Acids. 2024;5(2):201–220. 10.20517/evcna.2023.76 PMC11648467 39698535

[113] Xu G, Jin J, Fu Z, Wang G, Lei X, Xu J, Wang J. Extracellular vesicle-based drug overview: Research landscape, quality control and nonclinical evaluation strategies. Signal Transduct Target Ther. 2025;10(1):255. 10.1038/s41392-025-02312-w PMC12350758 40804047

[114] Martin JR, Cleary D, Abraham ME, Mendoza M, Cabrera B, Jamieson C, Marsala M, Ciacci JD. Long-term clinical and safety outcomes from a single-site phase 1 study of neural stem cell transplantation for chronic thoracic spinal cord injury. Cell Rep Med. 2024;5(12): Article 101841. 10.1016/j.xcrm.2024.101841 PMC11722094 39626671

[115] Guo W, Zhang X, Zhai J, Xue J. The roles and applications of neural stem cells in spinal cord injury repair. Front Bioeng Biotechnol. 2022;10: Article 966866. 10.3389/fbioe.2022.966866 PMC9465243 36105599

[116] Mou C, Xia Z, Wang X, Dai X, Wang J, Zhang C, Xu Y. Stem cell-derived exosome treatment for acute spinal cord injury: A systematic review and meta-analysis based on preclinical evidence. Front Neurol. 2025;16: Article 1447414. 10.3389/fneur.2025.1447414 PMC11802430 39926016

[117] Cipriano BP, Cunha RS, Santana TA, Silva KN, Loiola EC, Figueiredo JCQ, Silva EA, Lima AVR, Rossi EA, Silva IC, et al. Early intervention with extracellular vesicles derived from human umbilical cord mesenchymal stem cells enhances survival and functional recovery after spinal cord injury in rats. Front Pharmacol. 2025;16: Article 1695914. 10.3389/fphar.2025.1695914 PMC12646952 41311838

[118] Weidner N, Abel R, Maier D, Röhl K, Röhrich F, Baumberger M, Hund-Georgiadis M, Saur M, Benito-Penalva J, Rehahn K, et al. Safety and efficacy of intrathecal antibodies to Nogo-A in patients with acute cervical spinal cord injury: A randomised, double-blind, multicentre, placebo-controlled, phase 2b trial. Lancet Neurol. 2025;24(1):42–53. 10.1016/S1474-4422(24)00447-2 39706632

[119] Debnath K, Heras KL, Rivera A, Lenzini S, Shin JW. Extracellular vesicle-matrix interactions. Nat Rev Mater. 2023;8(6):390–402. 10.1038/s41578-023-00551-3 PMC10919209 38463907

[120] Tam NW, Becker A, Mangiarotti A, Cipitria A, Dimova R. Extracellular vesicle mobility in collagen I hydrogels is influenced by matrix-binding Integrins. ACS Nano. 2024;18(43):29585–29601. 10.1021/acsnano.4c07186 PMC11526431 39400273

[121] Hao D, Lu L, Song H, Duan Y, Chen J, Carney R, et al. Engineered extracellular vesicles with high collagen-binding affinity present superior in situ retention and therapeutic efficacy in tissue repair. Theranostics. 2022;12(13):6021–6037. 10.7150/thno.70448 PMC9373818 35966577

[122] Wang S, Wang R, Chen J, Yang B, Shu J, Cheng F, Tao Y, Shi K, Wang C, Wang J, et al. Controlled extracellular vesicles release from aminoguanidine nanoparticle-loaded polylysine hydrogel for synergistic treatment of spinal cord injury. J Control Release. 2023;363:27–42. 10.1016/j.jconrel.2023.09.026 37722419

[123] Guan P, Fan L, Zhu Z, Yang Q, Kang X, Li J, Zhang Z, Liu S, Liu C, Wang X, et al. M2 microglia-derived exosome-loaded electroconductive hydrogel for enhancing neurological recovery after spinal cord injury. J Nanobiotechnol. 2024;22(1):8. 10.1186/s12951-023-02255-w PMC10763283 38167113

[124] Deng M, Xie P, Xue H, Chen Q, Zhou Y, Ming J, et al. Decellularized tissue matrices hydrogels functionalized with extracellular vesicles promote macrophage reprogramming and neural stem cell differentiation for spinal cord injury repair. J Nanobiotechnol. 2025;23:139. 10.1186/s12951-025-03152-0 PMC11853540 40001048

[125] Dong X, Lu Y, Hu Q, Zeng C, Zheng J, Huang J, Dong H, Zou P, Wang T, Wu Y, et al. Engineered exosome-loaded silk fibroin composite hydrogels promote tissue repair in spinal cord injury via immune checkpoint blockade. Small. 2025;21(27): Article e2412170. 10.1002/smll.202412170 40384273

[126] Spiers JG, Vassileff N, Hill AF. Neuroinflammatory modulation of extracellular vesicle biogenesis and cargo loading. Neuromolecular Med. 2022;24(4):385–391. 10.1007/s12017-022-08704-3 PMC9684235 35181852

[127] Salvi V, Gaudenzi C, Mariotti B, Giongrandi G, Alacqua S, Gianello V, Schioppa T, Tiberio L, Ceribelli A, Selmi C, et al. Cell damage shifts the microRNA content of small extracellular vesicles into a toll-like receptor 7-activating cargo capable to propagate inflammation and immunity. Cell Commun Signal. 2024;22:536. 10.1186/s12964-024-01924-z PMC11545887 39516877

[128] Lu W, Du X, Zou S, Fang Q, Wu M, Li H, Shi B. IFN-gamma enhances the therapeutic efficacy of MSCs-derived exosome via miR-126-3p in diabetic wound healing by targeting SPRED1. J Diabetes. 2024;16(1): Article e13465. 10.1111/1753-0407.13465 PMC10809290 37646268

[129] Wang M, Li R, Sheng S, Dong Z, Bai L, Wang X, Wang J, Lai Y, Chen X, Gao J, et al. Combination therapy using intestinal organoids and their extracellular vesicles for inflammatory bowel disease complicated with osteoporosis. J Orthop Translat. 2025;53:26–36. 10.1016/j.jot.2025.05.008 PMC12167829 40525095

[130] Cho AN, Jin Y, An Y, Kim J, Choi YS, Lee JS, Kim J, Choi WY, Koo DJ, Yu W, et al. Microfluidic device with brain extracellular matrix promotes structural and functional maturation of human brain organoids. Nat Commun. 2021;12(1):4730. 10.1038/s41467-021-24775-5 PMC8342542 34354063

[131] Koch LF, Best T, Wustenhagen E, Adrian K, Rammo O, Saul MJ. Novel insights into the isolation of extracellular vesicles by anion exchange chromatography. Front Bioeng Biotechnol. 2023;11: Article 1298892. 10.3389/fbioe.2023.1298892 PMC10836363 38312509

[132] Ene J, Syed F, Ma S, Ma S, Joshi S, Li Y. Extracellular vesicle production from human blood vessel organoids in a vertical wheel bioreactor. Methods Mol Biol. 2025. 10.1007/7651_2025_661 PMC12497411 40762884

[133] Lorach H, Galvez A, Spagnolo V, Martel F, Karakas S, Intering N, Vat M, Faivre O, Harte C, Komi S, et al. Walking naturally after spinal cord injury using a brain-spine interface. Nature. 2023;618(7963):126–133. 10.1038/s41586-023-06094-5 PMC10232367 37225984

[134] Hu X, Li N, Pang M, Bai S, Mo J, Yao S, Lu Y, Huang M, Di J, Kang Y, et al. Brain-computer interface-controlled exoskeleton training for lower-limb rehabilitation in spinal cord injury: A pilot randomized clinical trial. Ann Neurol. 2026;99(5):1124–1138. 10.1002/ana.78144 41480666

[135] Manna I, De Benedittis S, Porro D. Extracellular vesicles in multiple sclerosis: Their significance in the development and possible applications as therapeutic agents and biomarkers. Genes (Basel). 2024;15(6):772. 10.3390/genes15060772 PMC11203165 38927708

[136] Simoes-Abade MBC, Patterer M, Nicaise AM, Pluchino S. Brain organoid methodologies to explore mechanisms of disease in progressive multiple sclerosis. Front Cell Neurosci. 2024;18: Article 1488691. 10.3389/fncel.2024.1488691 PMC11688374 39744673

[137] Zhao HH, Haddad G. Brain organoid protocols and limitations. Front Cell Neurosci. 2024;18: Article 1351734. 10.3389/fncel.2024.1351734 PMC10987830 38572070

[138] Yasuda S, Bando K, Henry MP, Libertini S, Watanabe T, Bando H, Chen C, Fujimori K, Harada K, Kuroda T, et al. Detection of residual pluripotent stem cells in cell therapy products utilizing droplet digital PCR: An international multisite evaluation study. Stem Cells Transl Med. 2024;13(10):1001–1014. 10.1093/stcltm/szae058 PMC11465167 39120125

[139] Nguyen TD, Chooi WH, Jeon H, Chen J, Tan J, Roxby DN, Lee CY, Ng SY, Chew SY, Han J. Label-free and high-throughput removal of residual undifferentiated cells from iPSC-derived spinal cord progenitor cells. Stem Cells Transl Med. 2024;13(4):387–398. 10.1093/stcltm/szae002 PMC11016845 38321361

[140] Watanabe T, Yasuda S, Chen CL, Delsing L, Fellows MD, Foldes G, Kusakawa S, Mouriès LP, Sato Y. International evaluation study of a highly efficient culture assay for detection of residual human pluripotent stem cells in cell therapies. Regen Med. 2023;18(3):219–227. 10.2217/rme-2022-0207 36852420

[141] Watanabe T, Yasuda S, Kusakawa S, Kuroda T, Furukawa H, Futamura M, Shimizu S, Morishita A, Hata S, Koeda A, et al. Multisite studies for optimization of a highly efficient culture assay used for in vitro detection of residual undifferentiated human pluripotent stem cells intermingled in cell therapy products. Regen Ther. 2024;26:315–323. 10.1016/j.reth.2024.06.007 PMC11231703 38983832

[142] Watanabe T, La Shu S, del Rio-Espinola A, Ferreira JR, Bando K, Lemmens M, Pande P, de Wolf C, Chen CL, Elke E, et al. Evaluating teratoma formation risk of pluripotent stem cell-derived cell therapy products: A consensus recommendation from the Health and Environmental Sciences Institute’s International Cell Therapy Committee. Cytotherapy. 2025;27(9):1072–1084. 10.1016/j.jcyt.2025.04.062 PMC12353191 40392167

[143] Maiers M, Sullivan S, McClain C, Leonhard-Melief C, Turner ML, Turner D. Harnessing global HLA data for enhanced patient matching in iPSC haplobanks. Cytotherapy. 2025;27(3):300–306. 10.1016/j.jcyt.2024.11.001 39718520

[144] Madrid M, Lakshmipathy U, Zhang X, Bharti K, Wall DM, Sato Y, Muschler G, Ting A, Smith N, Deguchi S, et al. Considerations for the development of iPSC-derived cell therapies: A review of key challenges by the JSRM-ISCT iPSC Committee. Cytotherapy. 2024;26(11):1382–1399. 10.1016/j.jcyt.2024.05.022 PMC11471376 38958627

[145] Syromiatnikova V, Prokopeva A, Gomzikova M. Methods of the large-scale production of extracellular vesicles. Int J Mol Sci. 2022;23(18): Article 10522. 10.3390/ijms231810522 PMC9506336 36142433

[146] Jakl V, Ehmele M, Winkelmann M, Ehrenberg S, Eiseler T, Friemert B, Rojewski MT, Schrezenmeier H. A novel approach for large-scale manufacturing of small extracellular vesicles from bone marrow-derived mesenchymal stromal cells using a hollow fiber bioreactor. Front Bioeng Biotechnol. 2023;11: Article 1107055. 10.3389/fbioe.2023.1107055 PMC9904364 36761296

[147] Wiest EF, Zubair AC. Generation of current good manufacturing practices-grade mesenchymal stromal cell-derived extracellular vesicles using automated bioreactors. Biology. 2025;14(3): Article 313. 10.3390/biology14030313 PMC11940689 40136569

[148] Korchak JA, Wiest EF, Zubair AC. How do we assess batch-to-batch consistency between extracellular vesicle products? Transfusion. 2023;63(2):279–287. 10.1111/trf.17156 36329638

[149] Verma N, Arora S. Navigating the global regulatory landscape for exosome-based therapeutics: Challenges, strategies, and future directions. Pharmaceutics. 2025;17:990. 10.3390/pharmaceutics17080990 PMC12389065 40871013

[150] Takakura Y, Hanayama R, Akiyoshi K, Futaki S, Hida K, Ichiki T, Ishii-Watabe A, Kuroda M, Maki K, Miura Y, et al. Quality and safety considerations for therapeutic products based on extracellular vesicles. Pharm Res. 2024;41:1573–1594. 10.1007/s11095-024-03757-4 PMC11362369 39112776

[151] Zhao S, Di Y, Fan H, Xu C, Li H, Wang Y, Wang W, Li C, Wang J. Targeted delivery of extracellular vesicles: The mechanisms, techniques and therapeutic applications. Mol Biomed. 2024;5(1):60. 10.1186/s43556-024-00230-x PMC11579273 39567444

[152] Ahn SJ, Lee S, Kwon D, Oh S, Park C, Jeon S, Lee JH, Kim TS, Oh IU. Essential guidelines for manufacturing and application of organoids. Int J Stem Cells. 2024;17(2):102–112. 10.15283/ijsc24047 PMC11170116 38764240

[153] Li Q, Li Y, Shao J, Sun J, Hu L, Yun X, Liuqing C, Gong L, Wu S. Exploring regulatory frameworks for exosome therapy: Insights and perspectives. Health Care Sci. 2025;4:299–309. 10.1002/hcs2.70028 PMC12371722 40861511

[154] Terai S, Asonuma M, Hoshino A, Kino-Oka M, Ochiya T, Okada K, Sato Y, Takahashi Y, Tobita M, Tsuchiya A. Guidance on the clinical application of extracellular vesicles. Regen Ther. 2025;29:43–50. 10.1016/j.reth.2025.02.008 PMC11930518 40124467

[155] Pincela Lins PM, Lintsen D, Willems S, Moya Gomez AM, Pirlet E, Schildermans K, Mertens I, Michiels L, Nelissen I, Bronckaers A. Toward standardization of upscaled manufacturing of extracellular vesicles in hollow-fiber bioreactors. Trends Biotechnol. 2026;44(3):862–880. 10.1016/j.tibtech.2025.10.010 41206316

[156] Mizenko RR, Feaver M, Bozkurt BT, Lowe N, Nguyen B, Huang KW, Wang A, Carney RP. A critical systematic review of extracellular vesicle clinical trials. J Extracell Vesicles. 2024;13(10): Article e12510. 10.1002/jev2.12510 PMC11428870 39330928

